# The role of intermediary representations in solving lever balance problems with different target representations and problem complexities

**DOI:** 10.3389/fpsyg.2026.1815951

**Published:** 2026-09-17

**Authors:** Cuiting Xie, Liyu Ye, Jin Chen, Wenting Hong, Zhuoyan Fang, Fei Guan, Jiameng Yao, Jianwen Xiong, Yang Xiao, Taorong Liu

**Affiliations:** 1 School of Physics, South China Normal University, Guangzhou, China; 2 Hetian Middle School of Luhe County, Shanwei, China; 3 School of Physics and Electronics, Nanning Normal University, Nanning, China

**Keywords:** cognitive load, intermediary representations, physics problem solving, problem complexity, target representation

## Abstract

**Introduction:**

Intermediary representations are widely regarded as potentially useful supports in physics problem solving, although their association with performance may vary across task characteristics. This study examined how intermediary representation use was associated with performance on lever balance problems and how this association varied across target representation types and problem complexity types.

**Methods:**

A total of 130 ninth grade students solved 12 problems representing three target representation types and four problem complexity types. Problem solving success was analyzed using a generalized linear mixed effects model.

**Results:**

Intermediary representation use was associated with higher overall problem solving success. However, this association was strongest for mathematical and tabular target representations and more limited for diagrammatic target representations. The magnitude of the association also varied across problem complexity types. Larger differences were observed for problems requiring the coordination of force magnitude with pivot location or lever arm length, whereas smaller or nonsignificant differences were observed for problems involving force magnitude only or force magnitude and direction.

**Discussion:**

These findings suggest that intermediary representation use may be most strongly associated with successful performance when problems require manageable relational coordination and translation into mathematical or tabular forms.

## 1 Introduction

Representations play a central role in physics problem solving, serving not only as vehicles for information display but also as epistemic tools that shape how learners interpret, reason about, and solve physical situations (Fredlund et al., 2014; Treagust et al., 2017). Verbal, graphical, and symbolic representations each foreground different aspects of physical meaning and impose distinct cognitive demands (Waldrip and Prain, 2012; Wu and Puntambekar, 2012). Consequently, representational format directly influences students’ solution strategies, error patterns, and overall problem-solving performance (Kohl and Finkelstein, 2006).

Recent work has drawn attention to the role of intermediary representations in mediating translation between source and target forms (Bossé et al., 2011; Chang et al., 2021; Kohl and Finkelstein, 2008). Rather than transforming information directly from one format to another, successful solvers often reorganize information into intermediate structures that better align with disciplinary conventions and solution demands. These intermediary constructions appear to support sense making, reduce structural mismatches, and preserve relational coherence during transformation (Bossé et al., 2011).

Despite growing recognition of their importance, two critical issues remain insufficiently examined. First, most empirical studies have investigated intermediary representations within constrained translation tasks, such as multiple-choice formats, which provide limited insight into how such representations are spontaneously constructed and used in constructed problem solving (Chang et al., 2021). Research on learner generated drawing further suggests that constructing external representations can support the selection, organization, and integration of information, although its effectiveness depends on task design, learner knowledge, and instructional guidance (Ainsworth et al., 2011; Fiorella and Zhang, 2018; van Meter and Garner, 2005; Wu and Rau, 2019). Second, relatively little is known about the conditions under which intermediary representation use is most strongly associated with successful performance. Representational demands vary across target representation types, and problem complexity further influences the extent to which information must be coordinated concurrently (Ibrahim et al., 2017; Lee and Heyworth, 2000). These considerations suggest that the role of intermediary representations may vary with task structure.

The present study addresses this issue by examining intermediary representations in lever balance problem solving across target representation types and problem complexity types, while maintaining consistent textual and pictorial problem entry conditions. The goal is to identify the task conditions across which the association between intermediary representation use and students’ problem solving success varies.

### 1.1 Representations and multiple representations in physics problem solving

From a cognitive perspective, representation can be understood as the outcome of information coding and processing, through which internal ideas or experiences are expressed in external forms (Bruner, 1964). More broadly, representations involve the external exhibition or presentation of events, phenomena, thoughts, or tangible objects, enabling learners to engage with ideas beyond purely internal mental processing (Wu and Puntambekar, 2012). Through representations, abstract entities and relations in physics become accessible for inspection, manipulation, and reasoning, thereby supporting sense making and problem solving.

In physics education research, representations are increasingly viewed not merely as means for presenting information, but as epistemic tools that actively shape how learners reason about physical situations and construct solutions (Fredlund et al., 2014; Treagust et al., 2017). Different representations foreground different aspects of physical meaning and invite different forms of inference. From this perspective, learning physics involves not only acquiring conceptual knowledge, but also developing competence in interpreting and using representations in ways that are aligned with disciplinary norms. Representations therefore play an active role in meaning making and provide an important window into students’ reasoning processes during problem solving (Fredlund et al., 2014).

In science and physics learning, three broad categories of representations are commonly distinguished: verbal textual, visual graphical, and symbolic mathematical representations (Waldrip and Prain, 2012; Wu and Puntambekar, 2012). Verbal textual representations use natural language to describe physical phenomena, concepts, and relations. Visual graphical representations include pictures, diagrams, graphs, tables, animations, and videos, which convey information through spatial and visual organization. Symbolic mathematical representations use symbols, signs, and equations to express physical principles and quantitative relationships in a compact and formalized manner (van Heuvelen and Zou, 2001; Wu and Puntambekar, 2012). This typology provides a useful basis for analyzing how different representational forms impose distinct cognitive and interpretive demands during physics problem solving.

Empirical research demonstrates that representational format is a consequential dimension of physics problem solving. Visual graphical representations often support qualitative reasoning by making relational structures salient, whereas symbolic mathematical representations enable precise calculation and formal manipulation. Verbal representations frequently provide contextual framing and conceptual explanation. However, these representations are not interchangeable. Studies comparing isomorphic problems presented in different representational formats consistently reveal systematic differences in students’ performance, solution strategies, and error patterns (Kohl and Finkelstein, 2006). Moreover, students tend to rely on familiar representations even when those representations are inefficient or poorly suited to the task at hand (De Cock, 2012). These findings indicate that representational format directly shapes how problems are interpreted, which strategies are activated, and how solutions are constructed.

Consistent with this epistemic view, research further shows that representations afford and constrain reasoning in distinct ways. From a disciplinary perspective, each representation foregrounds particular aspects of physical meaning while backgrounding others. Fredlund et al. (2014) introduced the notion of disciplinary affordance to capture this idea, arguing that representations differ in the kinds of disciplinary knowledge they make accessible to learners. Difficulties in problem solving may therefore arise when students fail to perceive or appropriately use these affordances. Empirical studies illustrate how representational constraints can lead to systematic reasoning difficulties. For example, Bollen et al. (2017) showed that university students often struggled to coordinate magnitude and direction information when working with symbolic and graphical representations of vector fields. These difficulties reflected deeper challenges in mapping physical meaning onto representational features, rather than merely procedural errors. Similarly, De Cock (2012) demonstrated that specific representational details can cue particular problem solving strategies, sometimes leading students toward unproductive solution paths. Together, these findings underscore that representations do not simply convey information, but actively structure how problems are understood and approached.

Given the complementary strengths of different representations, multiple representations are widely used in physics instruction and problem solving. A substantial body of research indicates that using multiple representations can enhance students’ science learning more effectively than relying on a single representation (Adadan, 2013; Adadan et al., 2009; Ainsworth, 2006; Schnotz and Bannert, 2003). By highlighting different features of the same phenomenon, multiple representations can support conceptual integration and promote deeper understanding (Kozma et al., 2000; Kozma, 2003; Waldrip and Prain, 2012; Yeo and Gilbert, 2014). Expert physicists, in particular, demonstrate flexibility in selecting and coordinating representations in response to task demands, a pattern that distinguishes them from novices (Kohl and Finkelstein, 2008).

At the same time, research has consistently documented challenges associated with the use of multiple representations. Coordinating information across representations imposes substantial cognitive demands, especially for learners with limited representational competence. Studies show that students often recognize individual representations yet struggle to integrate them effectively during problem solving (Campos et al., 2020). Moreover, representational difficulties are not uniform across formats. Campos et al. (2023) demonstrated that interpretation and coordination challenges depend partly on the target representation, indicating that representational fluency develops unevenly across different representational forms. Without appropriate instructional support, multiple representations may therefore increase cognitive load rather than alleviate it. Instructional research suggests that explicit guidance can foster more productive representational use (Kohl et al., 2007), while recent work cautions that representational overload can hinder learning when coordination demands are not carefully managed (Marshman et al., 2024).

### 1.2 Representation translation in physics problem solving

Representation translation refers to the cognitive process through which information encoded in a source representation is interpreted and reconstructed in a target representation (Janvier, 1987). Subsequent research has emphasized that translation involves extracting relevant relationships from a source representation and re expressing those relationships in accordance with the conventions and constraints of a target representation (Adu-Gyamfi et al., 2012; Keig and Rubba, 1993). Importantly, representational translation is conceptually distinct from representational recognition. Empirical studies demonstrate that recognizing a representation and translating it into another rely on different cognitive processes, with translation requiring deeper interpretive and constructive activity (Campos et al., 2020; Lesh et al., 1987). Successful representational translation therefore depends on identifying which features of the source representation are conceptually essential and determining how those features map onto the representational grammar of the target format (Ainsworth, 2006; van Heuvelen and Zou, 2001). This process imposes substantial cognitive demands, as learners must simultaneously interpret the source representation, suppress misleading surface features, and construct a coherent target representation, placing pressure on working memory and attentional control (Adu-Gyamfi et al., 2017; Janvier, 1987).

Beyond its cognitive demands, representational translation plays a direct functional role in physics problem solving performance. Many physics problems implicitly require translation even when only a single representation is explicitly presented. Solving such problems often involves inferring information that is not directly available in the given representation or reformulating the problem in a representation that better supports the solution process (De Cock, 2012; Larkin and Simon, 1987). When learners encounter unfamiliar or cognitively demanding representational formats, they may attempt to translate the problem into a more familiar representation to facilitate reasoning. However, such translations are frequently incomplete or incorrect, particularly when learners lack fluency with the representational conventions involved (Jones and Schkade, 1995). These findings underscore representational translation as a key mechanism linking representational format to problem solving success, rather than a peripheral skill applied only in explicitly multi representational tasks.

Research comparing novice and expert problem solvers reveals systematic differences in how representational translation is enacted during physics problem solving. Experts tend to translate selectively and strategically, choosing representations that align with task demands and solution goals rather than translating indiscriminately (Kohl and Finkelstein, 2008; Kozma and Russell, 1997). In contrast, novices often remain fixed within a single representation or engage in translation attempts driven primarily by surface familiarity rather than conceptual utility. Kozma and Russell (1997) showed that although novices and experts may perform similarly in selecting appropriate target representations, novices are less successful in constructing those representations in a physically meaningful way. Similarly, Kohl and Finkelstein (2008) found that experts exhibited greater representational flexibility, whereas novices’ translation behavior was more strongly constrained by surface features of representations. These results indicate that representational translation competence develops with expertise and constitutes an important dimension of physics problem solving proficiency.

Analyses of student work have further identified recurring error patterns that illuminate the sources of difficulty in representational translation. These errors can be broadly grouped into several categories. One common source of difficulty involves misinterpretation of the source representation, where students fail to extract relevant information due to incomplete understanding of representational conventions (Bollen et al., 2017; Keig and Rubba, 1993). A second source concerns failures to preserve relational structure during translation, resulting in target representations that appear superficially plausible but are physically incorrect (Adu-Gyamfi et al., 2012). In addition, students may correctly interpret the source representation yet commit procedural or implementation errors when constructing the target representation, particularly in mathematically symbolic forms that require fluency with formal operations (Adu-Gyamfi et al., 2012). Together, these findings suggest that translation errors reflect the interaction of representational understanding, conceptual knowledge, and procedural skill, rather than a single underlying deficit.

An important insight emerging from recent research is that representational translation difficulty is not uniform but depends on the specific source and target representations involved. Studies have shown that students’ performance varies systematically across translation tasks involving different representational pairs, even when the underlying physics content is held constant (Klein et al., 2017). Such results indicate that certain representation pairs impose greater cognitive demands than others, supporting the view that representational translation is representation dependent rather than a generalizable skill. This conclusion is reinforced by studies in electricity and magnetism, which show that students often recognize individual representations yet struggle to coordinate them during translation, particularly when graphical or field based representations serve as either the source or the target (Campos et al., 2020, 2023). Taken together, these findings highlight the importance of attending to the specific representational demands embedded in physics tasks and provide a strong rationale for examining how representational structure constrains translation processes and problem solving performance.

### 1.3 Intermediary representations in representational translation

An important refinement in research on representational translation is the recognition that translations between certain types of representations are often indirect and therefore require the involvement of intermediary representations. Early theoretical work emphasized that some representational pairs cannot be efficiently transformed in a single step, but instead benefit from intermediate forms that reorganize information into structures that are more compatible with the target representation (Janvier, 1987; Lesh et al., 1987). From this perspective, intermediary representations function as transitional cognitive tools that mediate translation by bridging mismatches in informational structure and representational conventions between source and target formats.

Within mathematics and science education, intermediary representations have been conceptualized as process oriented resources rather than additional representational endpoints. Bossé et al. (2011) articulated this role clearly by arguing that intermediary representations reduce cognitive difficulty associated with direct translation attempts, help preserve relational structure, and support meaning making during transformation. In this framework, constructing an intermediary representation allows learners to temporarily reorganize information in a form with more accessible affordances, thereby facilitating subsequent translation and reducing errors that stem from surface level mapping.

Empirical research in physics education provides evidence consistent with this functional account. Studies of expert problem solving show that successful solvers often construct qualitative representations, such as diagrams or graphs, before formal equations (Kohl and Finkelstein, 2006, 2008). Evidence from work energy contexts also indicates that engaging with qualitative representations before equation use can support meaningful interpretation of mathematical expressions (van Heuvelen and Zou, 2001). Similarly, research on free body diagrams suggests that appropriately constructed diagrams can support qualitative analysis and constraint identification before equation construction (Rosengrant et al., 2009).

More recent work has examined intermediary representations explicitly as a process level resource within representational translation. For example, analyses of students’ written solution paths in torque and rotation contexts show that translation tasks frequently involve force diagrams or equations as intermediary representations (Chang et al., 2021). Successful students tend to employ these intermediaries strategically to reorganize information before constructing target representations, whereas unsuccessful students often attempt direct translation or use intermediaries in fragmented or inappropriate ways. This evidence indicates that the presence of an intermediary representation alone does not ensure successful translation because its usefulness also depends on accurate interpretation and appropriate use.

Taken together, prior research suggests that intermediary representations can function as process level resources during indirect representational translation. By reorganizing information into a form that is more compatible with the target, they may help preserve relational structure, coordinate qualitative and quantitative information, and support the construction of more formal representations (Ainsworth et al., 2011; Bossé et al., 2011; Chang et al., 2021; Fiorella and Zhang, 2018; Wu and Rau, 2019). Their effectiveness, however, is likely to depend on whether learners can construct and coordinate these representations appropriately.

### 1.4 Problem complexity in physics problem solving

Problem complexity is widely recognized as a key determinant of students’ performance, strategy selection, and representational behavior in physics problem solving. At the same time, prior research has not converged on a single, unified definition of problem complexity. Instead, complexity has been conceptualized from multiple perspectives, reflecting different sources of demand embedded in problem structure and solution processes. This diversity suggests that problem complexity is best understood as a multidimensional construct, rather than as a single task attribute.

An influential attempt to link complexity with problem difficulty was offered by Lee and Heyworth (2000), who conceptualized problem complexity as the demand imposed by a task on the problem solver. Drawing on earlier work by Holzman et al. (1983), they distinguished between a processing dimension and a content knowledge dimension of problem difficulty. The processing dimension concerns the manipulation and coordination of information during problem solving, such as the amount of information that must be maintained and integrated in working memory. The content knowledge dimension refers to domain specific skills required by a problem, including facility with formal procedures and symbolic manipulation. Importantly, Lee and Heyworth (2000) argued that both dimensions are reflected in the structure of the problem itself and can often be inferred directly from the problem expression, rather than being defined solely by learner performance.

Consistent with this theoretical distinction, subsequent empirical research in mathematics and physics education has examined problem complexity from multiple perspectives, implicitly addressing both content related and processing related sources of complexity. One prominent line of research has focused on the mathematical structure of problems as a key contributor to complexity. Yang (2019), for example, treated mathematical complexity as a core, externally observable contributor to physics problem difficulty. In this framework, mathematical complexity is characterized by features such as the number of equations required, the level of abstraction involved in applying physics principles, and the sophistication of mathematical operations needed to implement those principles. Empirical analyses demonstrated that mathematical complexity, together with other task related factors, accounted for a substantial proportion of variance in students’ problem solving performance on authentic physics tasks. From this perspective, mathematical complexity reflects the cognitive load associated with managing mathematical representations and operations, rather than surface level computational difficulty alone.

A second line of research has emphasized complexity arising from information coordination demands, particularly whether multiple unknown variables must be processed concurrently or can be resolved sequentially. Heffernan and Koedinger (1997) documented what is known as the *composition effect*, showing that students’ performance on composed problems involving multiple unknowns was significantly worse than would be predicted from their performance on matched single unknown problems. This effect highlights that problem difficulty increases substantially when learners must integrate multiple relations at the same time, rather than solving for unknowns in a stepwise manner. Empirical support for this distinction in physics comes from work by Ibrahim et al. (2017), who examined students’ conceptual performance on synthesis physics problems. Their findings indicated that increased mathematical complexity did not significantly impair performance on sequential synthesis problems. In contrast, for simultaneous synthesis problems, greater mathematical complexity led to markedly poorer conceptual performance. Together, these findings suggest that problem complexity cannot be reduced to the number of unknowns alone, but critically depends on the extent to which information must be coordinated concurrently during problem solving. In this sense, information coordination demands constitute a concrete operationalization of the processing dimension of problem complexity articulated by Lee and Heyworth.

### 1.5 The current study

Although prior research has highlighted the importance of intermediary representations in physics problem solving, several gaps remain. First, while Chang et al. (2021) examined the role of intermediary representations in students’ representational translation processes in physics, their work relied primarily on multiple choice formats to probe translation performance. Such designs constrain students’ responses to predefined options and provide limited insight into how intermediary representations are spontaneously constructed and used in constructed problem solving. There is therefore a need for evidence that captures intermediary representations as process level resources in classroom relevant constructed response problem solving.

Second, prior research has paid limited attention to the representational conditions under which school physics problems are commonly encountered, particularly with respect to the disciplinary affordances made available at problem entry. In textbook and classroom settings, problems are most often presented in verbal textual form, frequently accompanied by pictorial diagrams (Kohl and Finkelstein, 2006; van Heuvelen and Zou, 2001). These forms primarily serve as source representations that provide initial access to relevant physical relations, rather than as target representations that students are expected to construct. When such source representations differ across tasks or conditions, the affordances initially available to learners may also differ, making it difficult to determine whether variations in intermediary representation use reflect target representation demands or differences in initial affordance structures. Consequently, limited systematic evidence is available concerning how intermediary representations operate when textual and pictorial problem formats are deliberately held constant.

Third, prior research has argued that some representational translations are indirect and may involve one or more intermediary representations, such as mathematical equations (Kohl and Finkelstein, 2006, 2008). This perspective implies that different target representations impose distinct processing demands, which may be related to how intermediary representations are constructed, coordinated, and transformed during problem solving. Problem complexity may alter these demands by increasing the number of interacting elements and constraints. However, existing research has not systematically examined whether the association between intermediary representation use and problem solving success varies across target representation types and problem complexity types.

To address these gaps, the present study investigates the role of intermediary representations in ninth grade students’ solution of lever balance problems. The study systematically varies target representation type and problem complexity within classroom relevant textual and pictorial problem formats and examines their interaction with intermediary representation use.

*RQ1*: To what extent is intermediary representation use associated with students’ problem solving success in lever balance problems?

*RQ2*: How does the association between intermediary representation use and students’ problem solving success in lever balance problems vary across problem complexity types and target representation types?

## 2 Materials and methods

This study was conducted within the context of compulsory lower secondary physics education in mainland China. Lever balance is a core topic in the national physics curriculum standards and is typically introduced in Grade 8 as part of the unit on simple machines and mechanical equilibrium, with learning objectives emphasizing conceptual understanding of force and torque, proportional reasoning, experimental inquiry, and the use of multiple representations (Ministry of Education of the People’s Republic of China, 2022). In commonly used physics textbooks, such as those published by People’s Education Press, lever balance is presented through a combination of diagrammatic representations illustrating forces and lever arms, mathematical formulations of equilibrium conditions, and tabular representations for organizing experimental data (Peng, 2025). Classroom instruction and textbook tasks therefore require students to interpret and coordinate information across these representational forms. The present study was designed to align with this instructional context by employing lever balance problems and representation formats that closely reflect standard curriculum expectations and textbook practices.

### 2.1 Participants

Participants were ninth-grade students from three classes at a public junior high school in a city in southern China. The selection of ninth-grade students was based on two considerations. First, students at this grade level had already received systematic instruction on lever balance and torque equilibrium, ensuring that they possessed the necessary physics content knowledge for the tasks. Second, the test materials involved geometric reasoning related to special triangles and basic trigonometric relationships, which students had previously learned in their mathematics curriculum.

All classes were taught by the same physics teacher, ensuring consistency in instructional content and teaching approach. All 152 students enrolled in the three classes were invited to participate. The three classes exhibited comparable levels of physics achievement, as indicated by their mean scores on a recent standardized mock examination (M = 61.25, 61.32, and 61.39, respectively), with no statistically significant differences observed among the classes. Participation and submission of the test paper were voluntary. A total of 130 students submitted anonymous test papers, corresponding to a submission rate of 85.5%. Because the test papers were collected anonymously, they could not be linked to individual demographic information or achievement records. It was therefore not possible to compare students who submitted papers with those who did not at the individual level. These class level means should not be interpreted as achievement estimates specifically for the 130 students included in the analysis. Before data collection, informed consent was obtained from the students and their legal guardians.

### 2.2 Materials

The present study carefully designed an instrument to examine students’ problem solving performance across different representational formats and problem complexity types. This structure enabled systematic comparison across the 12 administered items, but it did not separate the intended condition factors from item specific features because each target representation by problem complexity cell was represented by one item. In what follows, the design of the materials and instrument is described in three components: (a) the design of target representations, (b) the design of problem complexity, and (c) the overall construction of the test instrument.

#### 2.2.1 Target representation design

Table 1 presents an exemplary item illustrating the unified source representation and its corresponding target representations used in the study. All lever balance problems were initially presented using a unified source representation that combined verbal descriptions with pictorial illustrations. This format reflects common presentation practices in physics textbooks and classroom instruction. Based on this shared source representation, students were required to solve each problem using one of three target representations: force diagram, tabular representation, or mathematical representation.

**Table 1:** Source representation and target representations of an exemplary lever balance item.

Representation	Description
Source	
Verbal + Pictorial	A light lever with pivot O. OA = 30 cm, OB = 10 cm. A load of 20 N is hung at point A. A downward effort force F is applied at point B to keep the lever horizontal. Find *F*. 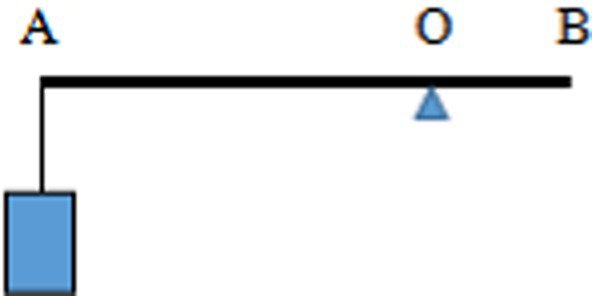
Target	
Force diagram	A free body diagram illustrating the lever, pivot O, the effort force F , the load force G , and the directions of all forces acting on the lever. 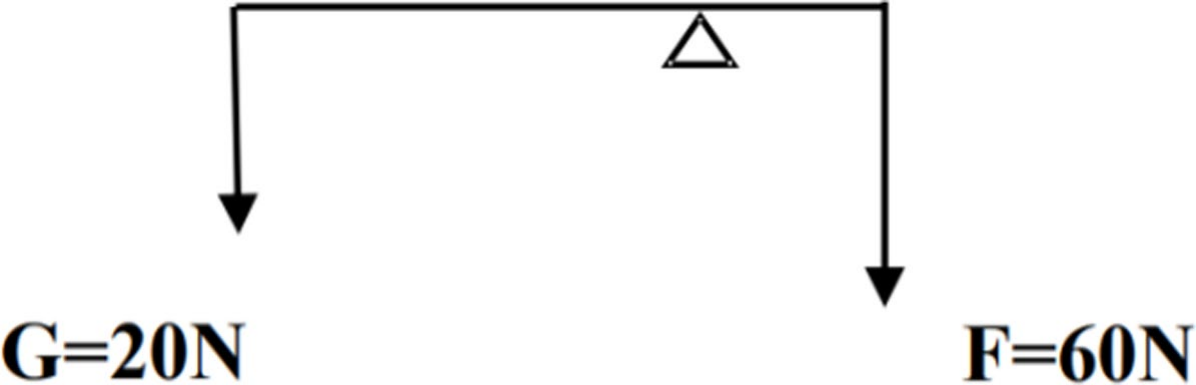
Tabular	A table organizing the relevant physical quantities involved in the problem. 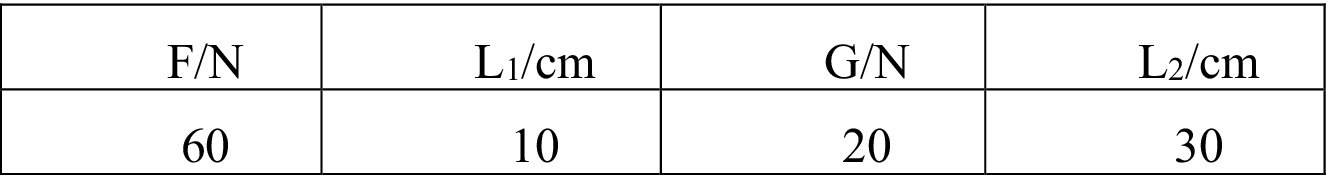
Mathematical	The equilibrium condition is expressed symbolically as F×OB=OA×G. Substituting known values yields F=(20N×30cm)/10cm=60N .

The force diagram representation required students to construct a free-body diagram showing the forces acting on the lever system. Key elements, including the pivot point, effort force, load force, force directions, and points of application, needed to be explicitly identified and represented using arrows or simplified line symbols. This representation emphasizes visual–spatial organization of forces and is commonly used to support force analysis in mechanics.

The tabular representation organized relevant physical quantities in a structured table. Typical entries included the effort force (*F*_₁_, in newtons), effort arm length (*L*_₁_, in centimeters), load force (*G*, in newtons), and load arm length (L₂, in centimeters). By systematically arranging variables, the table format was intended to make relationships among quantities explicit and support coordination of multiple variables in lever balance reasoning.

The mathematical representation required students to express the lever balance condition symbolically and perform numerical calculations using equations. Students applied the lever equilibrium principle, $F_{1}\times L_{1}=G\times L_{2}$ , and substituted known values to solve for the target force magnitude. This representation emphasizes symbolic manipulation and mathematical modeling of physical relationships.

#### 2.2.2 Problem complexity design

Problem complexity was operationalized according to both the number and the nature of unknown variables embedded in each lever balance problem, as well as the reasoning processes required to infer those unknowns. This operationalization was grounded in theoretical and empirical work suggesting that problem complexity reflects content related demands associated with unknown structures, as well as processing demands associated with information coordination and mental simulation during problem solving (Heffernan and Koedinger, 1997; Holzman et al., 1983; Ibrahim et al., 2017; Lee and Heyworth, 2000; Yang, 2019).

All problems shared the same ultimate goal of determining force magnitude using the equilibrium condition $F_{1}L_{1}=F_{2}L_{2}$ . However, they differed in the number and type of intermediate quantities that had to be inferred before this relation could be applied. These intermediate quantities included force direction, lever arm length, and pivot location. Based on these considerations, four types of problems with distinct complexity were designed (see Table 2).

**Table 2:** Examples of four types of lever balance problems with different types of complexity.

Type	Key unknown information	Example Item		Complexity	
				Hypothesized	Expert Rated
1	Force magnitude only	1. As shown in the figure, a light lever OB can rotate about point O. The length OA is 40 cm and OB is 60 cm. A load of 20 N is suspended at point B. A vertical upward effort force F is applied at point A. The lever is in horizontal equilibrium. Determine the magnitude of the force F .	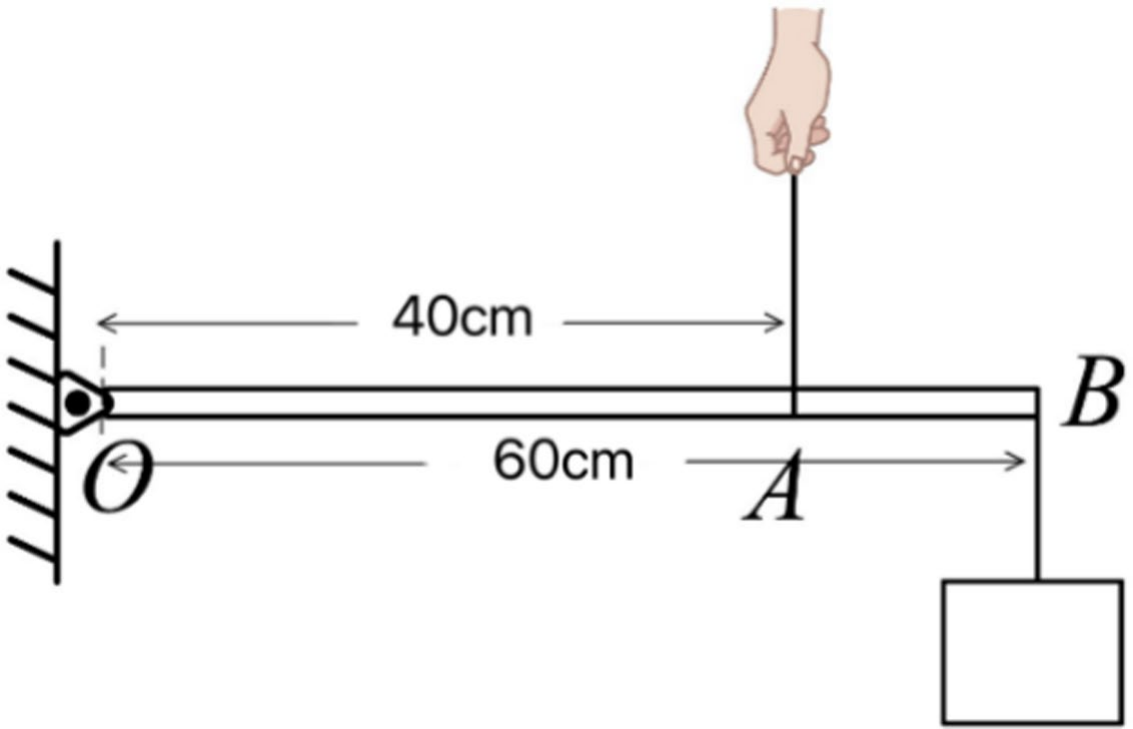	Low	2.14
2	Force magnitude and lever arm	2. As shown in the figure, a light lever AC is inclined at 30° to the horizontal, with A as the pivot. A load of 20 N is suspended at point B. A force F , perpendicular to the lever, is applied at point *C*. The lengths AB and AC are 20 cm and 60 cm, respectively. The lever is in equilibrium. Determine the magnitude of the force F .	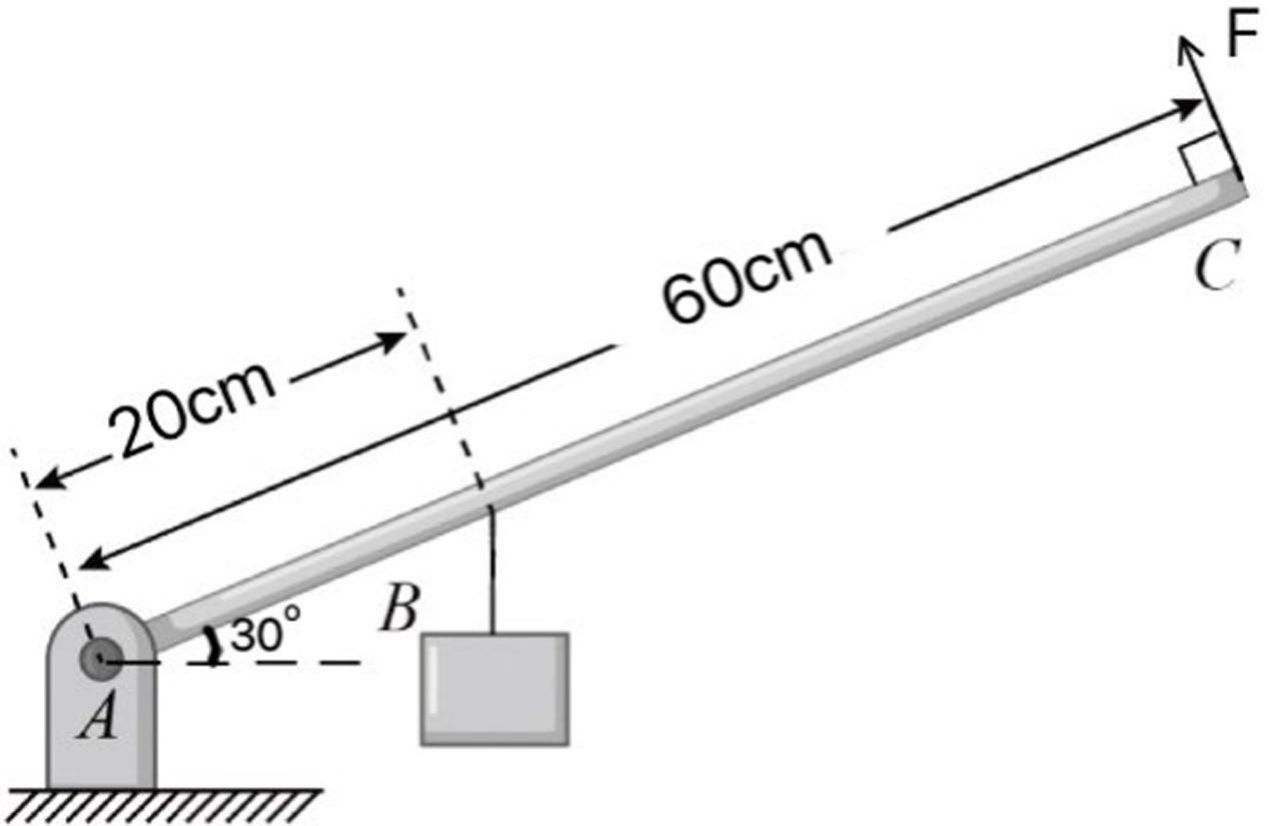	Lower-moderate	3.00
3	Force magnitude and pivot location	3. As shown in the figure, the section ABC of a piston pump can be regarded as a lever. During pumping, both the load force and the applied hand force act vertically. When a force $F_{1}=20$ N is applied at point A, the lever remains in equilibrium. Determine the magnitude of the load force $F_{2}$ .	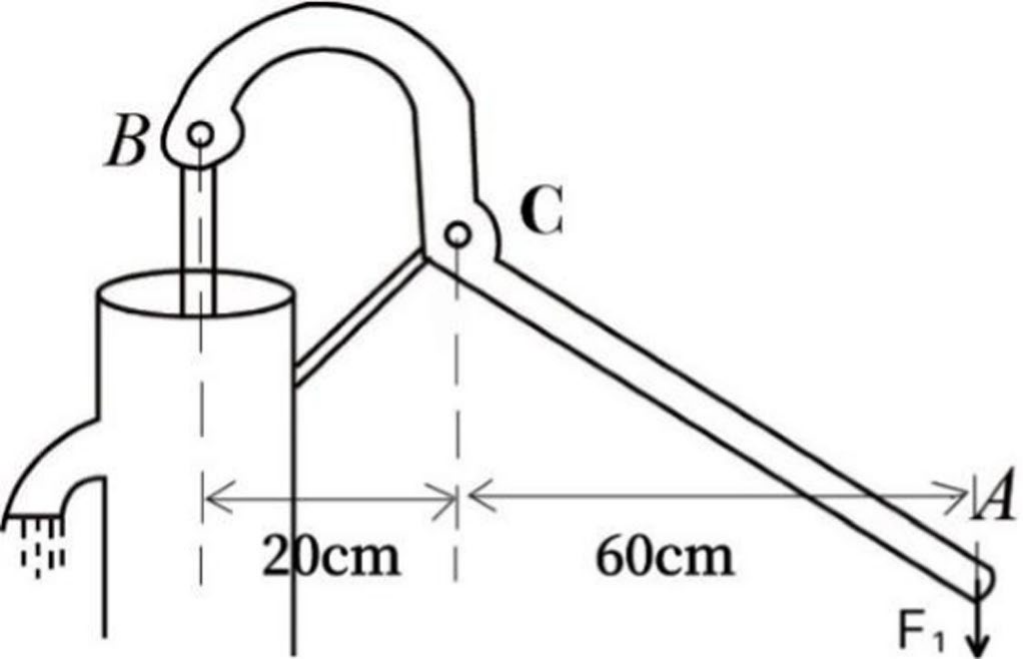	Upper-moderate	3.33
4	Force magnitude and direction	4. As shown in the figure, the section OBC of a side cutter can be regarded as a lever with O as the pivot. When a vertical downward effort force $F_{1}=50$ N is applied at point *C*, the lever remains in equilibrium. Determine the magnitude of the force exerted by the object on point B.	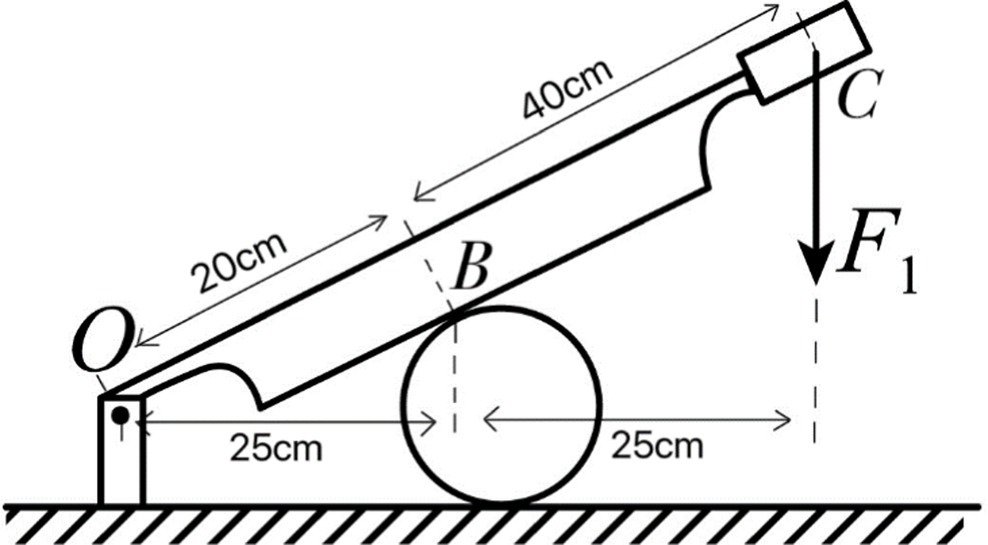	High	3.76

Type 1 involved a single unknown variable, the magnitude of either the effort force or the load force. All other relevant elements were explicitly provided and could be directly extracted from the given representation (see Item 1 in Table 1 as an example). This type required minimal information coordination and was treated as the low complexity condition.

The remaining three types each involved multiple unknown variables, but imposed different reasoning demands. Type 2 required determining both force magnitude and lever arm. Although force directions were provided, students had to determine the correct moment arm using geometric relations, sometimes involving special triangles or trigonometric reasoning. Research on torque and rotational equilibrium has shown that many students fail to reliably identify the perpendicular moment arm and instead rely on the radial distance from the pivot when reasoning about torque (Rimoldini and Singh, 2005). Such a misconception can compromise subsequent computation, increasing both conceptual and mathematical demands. However, once the correct lever arm is identified, the solution proceeds directly to magnitude calculation. For this reason, this type was categorized as lower moderate complexity.

Type 3 required determining both force magnitude and pivot location. Taking Item 3 in Table 2 as an example, the section ABC of the piston pump forms a bent lever system in which the rotational center is not explicitly indicated. To solve the problem, students must infer which joint remains stationary during operation and therefore functions as the rotational axis. This process relies on mental simulation of rotational tendency and evaluation of constraint relations. Research on mechanical reasoning suggests that mental simulation is a common and often effective basis for inferring motion and constraint relations (Hegarty, 2004). However, pivot identification affects the definition of both lever arms simultaneously. An incorrect pivot inference alters the entire geometric configuration and propagates through subsequent calculations. This structural dependency increases information coordination demands relative to Type 2, as multiple relational elements must be reorganized before applying equilibrium. Therefore, this type was categorized as upper moderate complexity.

Type 4 required determining both force magnitude and force direction. In these items, defining the relevant lever arm depends on correctly identifying the line of action of the unknown force. Thus, solvers must first infer force direction, often by reasoning about contact constraints, then determine which distance constitutes the lever arm, and only then apply equilibrium to compute magnitude. This layered inferential dependency increases information coordination demands, because an error at the direction stage prevents correct arm identification and propagates into the final magnitude calculation. Moreover, inferring contact force direction draws on conceptual knowledge that may be less directly practiced within lever equilibrium routines (Minstrell, 1992). For these reasons, Type 4 was hypothesized to be the highest complexity condition among the three unknown types.

To examine whether the hypothesized complexity types were empirically distinguishable, seven experienced physics teachers independently rated the 12 problems on a five point ordinal scale ranging from 1 (low complexity) to 5 (high complexity). The mean ratings were 2.14 (*SD* = 0.84), 3.00 (*SD* = 0.67), 3.33 (*SD* = 0.58), and 3.76 (*SD* = 0.85) for Types 1 to 4. The item level ratings were analyzed using a cumulative link mixed effects model with a proportional odds logit link, with problem type as a categorical fixed effect and random intercepts for item and rater. The inclusion of item as a random effect was necessary because each complexity type contained three distinct problems (see Overall Test Design). Treatment coding was used with Type 1 as the reference category. The model was estimated by maximum likelihood using the Laplace approximation and converged successfully. The analysis revealed a significant main effect of problem type, *χ*^2^(3) = 23.96, *p* < 0.001, suggesting that perceived complexity differed across types. Holm adjusted comparisons indicated that Type 1 was rated significantly lower than Type 2, *OR* = 17.53, *p* = 0.006; Type 3, *OR* = 44.38, *p* < 0.001; and Type 4, *OR* = 135.21, *p* < 0.001. Type 4 was also rated significantly higher than Type 2, *OR* = 7.72, *p* = 0.045, whereas the remaining comparisons were not significant. Thus, the ratings support a broad distinction between Type 1 and the multiple unknown types. Among the latter, Type 4 was rated significantly higher than Type 2, while the mean for Type 3, although not significantly different from either, fell between them as expected. Accordingly, the expert ratings provide partial empirical support for the proposed complexity categorization.

#### 2.2.3 Overall test design

Based on the 3 by 4 factorial design crossing target representation type and problem complexity type, a paper and pencil test consisting of 12 lever balance problems was constructed. Each target representation type was paired with each problem complexity type exactly once, resulting in a balanced set of items covering all condition cells. Because each of the 12 specific combinations was represented by one item, item identity coincided with the corresponding target representation by problem complexity cell.

Before completing the test, students were provided with explicit instructions explaining that physics problems can be represented in multiple ways, including verbal descriptions, diagrams, tables, and equations. An example problem was presented to illustrate how a single source representation could be transformed into different target representations. Students were informed that solving the subsequent problems might require translating between representations. They were encouraged to use scratch work freely and to record any additional representations they used during problem solving directly on the test paper. If no additional representations were used, no response was required in that space.

This design allowed intermediary representation use to emerge spontaneously during problem solving rather than being externally imposed. It therefore captured students’ written representational choices under classroom assessment conditions while enabling systematic analysis of representational behavior. The internal consistency reliability of the 12 item test, as estimated by Cronbach’s alpha, was 0.714, indicating acceptable reliability for research purposes.

### 2.3 Procedure

The test was administered to students in their classrooms during a regular, quiet self study period lasting approximately 40 min. With the assistance of the class teacher, one member of the research team explained the purpose of the study and the task requirements to the students. Before the test began, students were given the opportunity to ask questions about the instructions, which were addressed collectively to ensure a shared understanding of the task requirements.

After confirming that all students understood the instructions, the test commenced. Students completed the test individually under conditions closely resembling a formal classroom examination. No further questions were answered during the testing session. All test papers were submitted anonymously. All 130 submitted test papers contained complete and valid data and were retained for subsequent analysis.

### 2.4 Coding procedure and reliability

Problem solving success and intermediary representation use were coded independently at the item level. Problem solving success was coded dichotomously as successful (1) or unsuccessful (0). A response was coded as successful when it accurately completed the designated target representation and correctly expressed the lever balance relations relevant to that target. For mathematical target items, this required a correct equilibrium relation and final force magnitude. For tabular target items, the required forces and lever arms had to be correctly entered. For force diagram target items, the pivot, force directions, and relevant moment arms had to be correctly represented. Responses containing conceptual, representational, geometric, or computational errors, as well as responses that omitted required target elements, were coded as unsuccessful.

Intermediary representation use was coded dichotomously as present (1) or absent (0) based on students’ written work on the test paper. More precisely, the coded variable indexed the observable presence of an identifiable, task relevant, non-target representation. It was coded as present when a student spontaneously produced an optional external representation that differed from the designated target representation. The coding was therefore relational to the designated target rather than determined solely by representational form. For example, an equation could be coded as an additional representation when the target was diagrammatic or tabular, but not when the target was mathematical. Similarly, an additional force diagrammatic representation could be coded when the target was mathematical or tabular, but not when a force diagram was itself required. Because every item included a supplied schematic, students’ generated force diagrammatic information could appear either as a separately drawn force diagram or as force labels, distance annotations, lines of action, moment arms, pivot locations, or auxiliary geometric constructions added directly to the supplied schematic. The supplied schematic itself was not counted as an additional representation. When a force diagram was the designated target, such student generated force information was treated as part of the required response rather than as an intermediary representation. Simple copying, circling, underlining, isolated numerical work, illegible or unrelated marks, and clearly abandoned or crossed out work were not counted. Moreover, partial or incorrect additional representations were coded as present when an identifiable, task relevant representational structure was evident. Their accuracy or completeness did not determine the binary intermediary representation code. Consequently, a response could be unsuccessful while still being coded as using an intermediary representation. Conversely, a response could be successful without using one. Because the coding was based on the final written product, it did not establish that the additional representation was generated before the target response, mediated a transition between representations, or functioned productively during problem solving. When the designated target was incomplete, incorrect, or absent, the code indicated only the observable presence of an additional non-target representation and was not interpreted as evidence of a completed intermediary sequence. Supplementary Figure S1 provides concrete examples of successful and unsuccessful responses with and without intermediary representation use, including a partially useful construction followed by an arithmetic error and an incorrect intermediary representation. Supplementary Table S1 reports the distribution of the observed additional representation types across target conditions.

This coding approach is consistent with Chang et al. (2021), who identified whether intermediary representations were adopted rather than rating their accuracy, completeness, or sophistication on a polytomous scale. It was also appropriate to the present research question, which concerned whether students constructed an intermediary representation. The extent of externalization does not necessarily provide a direct index of cognitive or problem solving function, because learners with stronger prior knowledge may rely more extensively on internal schemas (Kalyuga et al., 2003).

Two raters independently applied these rules to all 1,560 item level responses. For problem solving success, percentage agreement was 94%, Cohen’s *κ* = 0.877, *z* = 34.648, *p* < 0.001. For intermediary representation use, percentage agreement was 95%, Cohen’s *κ* = 0.865, *z* = 34.147, *p* < 0.001. Following the independent coding, all disagreements were discussed by the two raters and resolved through consensus. The consensus codes were used in the subsequent analyses.

### 2.5 Data analysis

Given the repeated measures structure of the data, with multiple problems nested within individual students, a mixed effects modeling approach was adopted for analysis. This approach allows for the simultaneous examination of item level predictors while accounting for individual differences in overall performance. In addition, mixed effects models are well suited for handling unbalanced observations and variability across participants, which are common in classroom based assessment data (Theobald, 2018).

Because the outcome variable was binary, a generalized linear mixed effects model with a binomial distribution and logit link function was fitted to the data. Problem solving success served as the dependent variable. An initial model included intermediary representation use, target representation type, and problem complexity type, all two way interactions, and the three way interaction among these predictors. The three way interaction was not significant, *χ*^2^(6) = 1.228, *p* = 0.975, and was therefore excluded to obtain a more parsimonious model. The resulting model included the three main effects and all three two way interactions: Success ∼ IR + Complexity + Target + IR × Complexity + IR × Target + Complexity × Target + (1 | Participant), where IR represents intermediary representation use.

All categorical predictors were coded using centered simple contrasts. Responses without intermediary representation use, problems involving force magnitude only, and diagrammatic target representations served as the respective comparison categories. For intermediary representation use, responses without and with intermediary representations were coded −0.50 and 0.50, respectively. For problem complexity, each of the three more complex conditions was contrasted with the force only condition using centered coefficients. For target representation type, tabular and mathematical representations were each contrasted with diagrammatic representation using centered coefficients. Because the contrasts were centered, the intercept represented the expected log odds at the centered levels of the predictors rather than the expected log odds for one particular condition. Correspondingly, each lower order term was evaluated at the centered levels of the other categorical predictors.

Alternative random effects specifications were examined to determine whether participant and item variability could be estimated simultaneously. Because the interaction between target representation type and problem complexity defined the same 12 cells as the 12 item identities, this interaction was temporarily omitted from the diagnostic models. A crossed model containing participant and item random intercepts did not converge and was nearly unidentifiable. More complex specifications allowing the association with intermediary representation use to vary across items produced singular or nonconvergent solutions. In separate convergent models, the participant intercept variance was 1.066, corresponding to an ICC of 0.245, whereas the item intercept variance was 0.100, corresponding to an ICC of 0.030. Because these variance components were obtained from separate models, they were treated as diagnostic information rather than as a formal decomposition of participant and item variability. Given the repeated observations within participants, the substantial participant clustering, and the inability to estimate participant and item components reliably in the same model, only the participant random intercept was retained in the final model. The absence of an item random component was treated as an analytical limitation rather than as evidence that item variability was absent.

To distinguish within student from between student associations, we conducted a sensitivity analysis using a person mean decomposition of intermediary representation use. For each participant, the mean proportion of items containing an additional representation was calculated and centered around the sample mean to represent the between student component. The within student component was calculated by subtracting the participant mean from the binary item level intermediary representation code. The sensitivity model included both components as fixed predictors, item identity as a fixed factor, and a participant random intercept: Success ∼ IRWithin + IRBetween + Item + (1 | Participant). Including item identity as a fixed factor adjusted for differences among the 12 administered items without estimating an item random component. An algebraically equivalent model replacing the within student deviation with the original item level binary code was fitted to test whether the within student and between student coefficients differed. In this parameterization, the coefficient for the centered participant mean represented the difference between the two associations. Model convergence and dispersion were examined before interpreting the results.

The model was estimated by maximum likelihood using the Laplace approximation through the *glmer* function in the *lme4* package. Convergence and potential overdispersion were examined before the fixed effects were interpreted. Fixed effects were evaluated using omnibus Wald chi square tests. Pairwise comparisons are reported as odds ratios, together with their standard errors, *z* values, and adjusted *p-*values. Significant interactions were examined using estimated marginal means. For *post-hoc* comparisons involving multilevel factors, *p*-values were adjusted using the Tukey correction to control for familywise error rate. Analyses were conducted in *jamovi* (Version 2.6) using the *GAMLj* module, which interfaces with *R* (Version 4.4).

## 3 Results

Supplementary Table S1 presents the distribution of coded additional representation types across target representation conditions. Among the 520 responses requiring a force diagram, 168 responses, 32.3%, contained an additional mathematical representation. Among the 520 responses requiring a mathematical representation, 68 responses, 13.1%, contained an additional force diagrammatic representation. Among the 520 responses requiring a tabular representation, 32 responses, 6.2%, contained an additional force diagrammatic representation, while 94 responses, 18.1%, contained an additional mathematical representation. No additional tabular representations were observed. These distributions reflect the target relative coding rule and show that the composition of the intermediary representation category differed across target conditions.

Descriptive statistics for problem solving success across intermediary representation use, problem complexity type, and target representation type are presented in Table 3. The final generalized linear mixed effects model converged without warnings. Its conditional *R*^2^, representing variance accounted for by the fixed and random effects together, was 0.589, whereas the marginal *R*^2^, representing variance accounted for by the fixed effects, was 0.442. The participant intercept variance was 1.181, SD = 1.087, corresponding to an ICC of 0.264. A likelihood ratio test supported retaining the participant random intercept, *χ*^2^(1) = 100.331, *p* < 0.001. The dispersion ratio was 0.917, indicating no evidence of overdispersion. All estimated coefficients and confidence intervals were finite. Thus, although success rates approached the ceiling or floor in some conditions, they did not prevent convergence or produce infinite parameter estimates.

**Table 3:** Group sizes and problem-solving success rates by intermediary representation use, target representation, and problem complexity (standard deviations in parentheses).

Condition	IR absent		IR present		Combined success rates
	*n*	Success rates	*n*	Success rates	
Problem complexity type					
Force magnitude only	290	0.876 (0.330)	100	0.980 (0.141)	0.903 (0.297)
Force magnitude and lever arm	284	0.261 (0.440)	106	0.642 (0.482)	0.364 (0.482)
Force magnitude and pivot location	310	0.419 (0.494)	80	0.850 (0.359)	0.508 (0.501)
Force magnitude and direction	314	0.510 (0.501)	76	0.447 (0.501)	0.497 (0.501)
Target representation					
Tabular	394	0.589 (0.493)	126	0.825 (0.381)	0.646 (0.479)
Mathematical	452	0.535 (0.499)	68	0.882 (0.325)	0.581 (0.494)
Force diagram	352	0.409 (0.492)	168	0.619 (0.482)	0.477 (0.500)
Overall	1,198	0.516 (0.500)	362	0.740 (0.439)	0.568 (0.496)

Success rates are presented as proportions, with standard deviations in parentheses. The symbol *n* indicates the number of item level responses in each intermediary representation group. IR absent indicates that no additional non-target representation was observed, whereas IR present indicates that at least one additional non-target representation was observed.

Table 4 presents the results of the generalized linear mixed effects model. Significant main effects were observed for intermediary representation use, target representation type, and problem complexity type. Intermediary representation use was associated with differences in problem solving success, *χ*^2^(1) = 41.06, *p* < 0.001. The observed success rate was 0.740 for responses with intermediary representation use and 0.516 for responses without it. Problem complexity type also showed a significant main effect, *χ*^2^(3) = 98.88, *p* < 0.001. Problems involving force magnitude only had the highest observed success rate, 0.903, whereas observed success rates for the three multiple unknown types ranged from 0.364 to 0.508. A significant main effect was further found for target representation type, *χ*^2^(2) = 47.02, *p* < 0.001. Inspection of the corresponding success rates suggests that tabular and mathematical target representations were accompanied by higher success rates than force diagram representations.

**Table 4:** Fixed effects from the generalized linear mixed-effects model predicting problem-solving success.

Fixed effect	*χ* ^2^	df	*p*
Intermediary representation	41.06	1	<0.001
Target representation	47.02	2	<0.001
Problem complexity	98.88	3	<0.001
Target representation × problem complexity	43.03	6	<0.001
Intermediary representation × target representation	9.37	2	0.009
Intermediary representation × problem complexity	35.96	3	<0.001

In addition to these main effects, all two-way interactions were significant. These included the interaction between intermediary representation use and problem complexity type, *χ*^2^(3) = 35.96, *p* < 0.001, the interaction between intermediary representation use and target representation type, *χ*^2^(2) = 9.37, *p* = 0.009, and the interaction between target representation type and problem complexity type, *χ*^2^(6) = 43.03, *p* < 0.001. These interactions were examined in detail below.

### 3.1 Problem solving success across intermediary representation use and target representation types

To explore the interaction between intermediary representation use and target representation type, students’ problem solving success was grouped by target representation type and collapsed across problem complexity types. Figure 1 illustrates success rates across target representations with and without intermediary representation use. When intermediary representations were not used, Tukey adjusted pairwise comparisons showed that problems requiring tabular representations were associated with significantly higher success rates than problems requiring force diagram representations, *OR* = 3.18, 95% CI [2.07, 4.90], *SE* = 0.70, *z* = 5.25, *p* < 0.001, and problems requiring mathematical representations, *OR* = 1.87, 95% CI [1.24, 2.81], *SE* = 0.39, *z* = 3.00, *p* = 0.033. Problems requiring force diagram representations were associated with lower estimated odds of success than those requiring mathematical representations, but the difference was not significant, *OR* = 0.59, 95% CI [0.41, 0.85], *SE* = 0.11, *z* = −2.80, *p* = 0.057.

**Figure 1:**
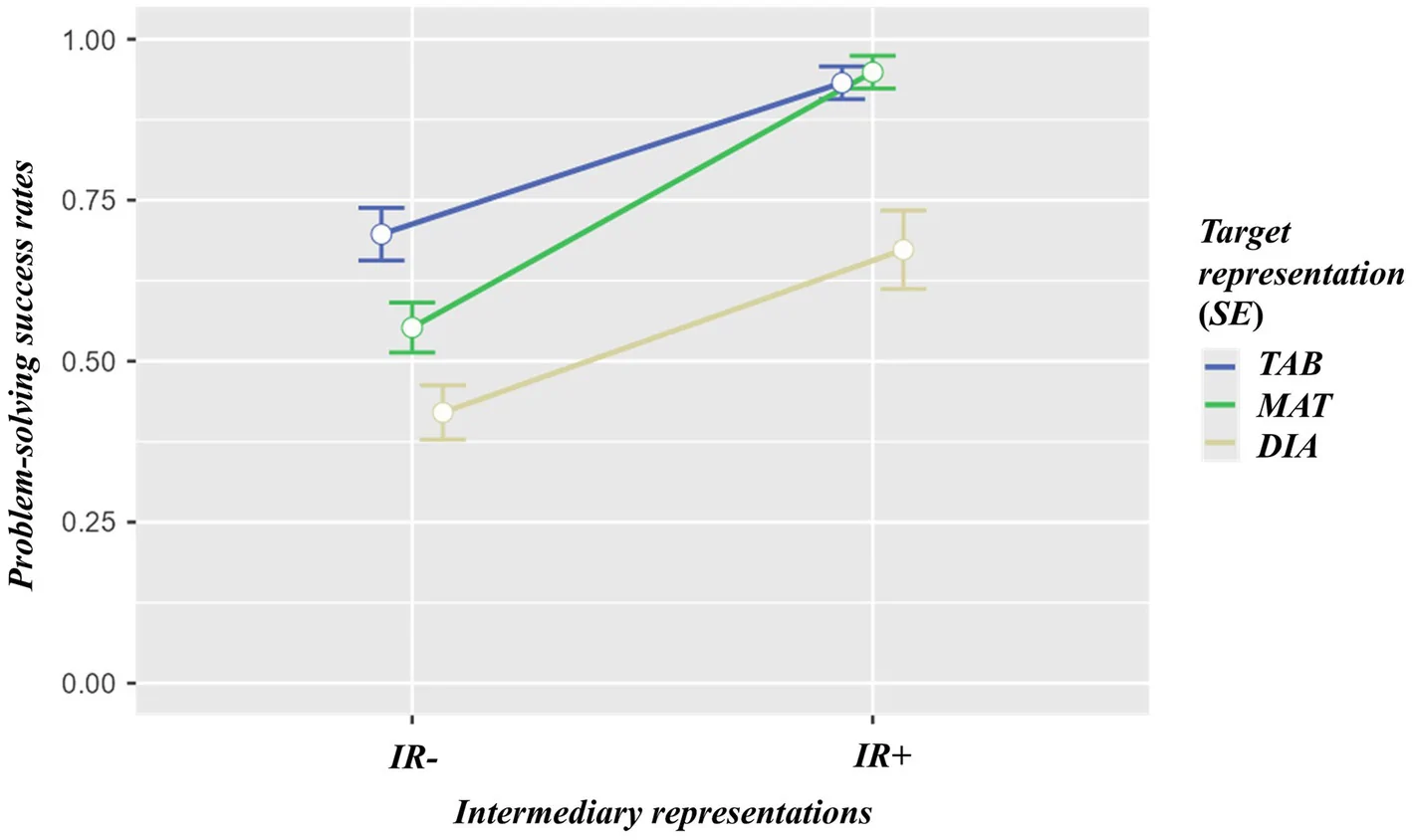
Problem-solving success rates across target representations with and without intermediary representations. TAB = tabular representation; MAT = mathematical representation; DIA = force diagram representation. IR− indicates no intermediary representation was used; IR+ indicates intermediary representation use. Error bars represent ±1 standard error.

Intermediary representation use was significantly associated with higher success rates across all target representation types, as shown in the lower panel of Table 3. The largest association was observed for mathematical target representations, for which intermediary representation use was associated with markedly higher success rates (*OR* = 15.06, 95% CI [5.28, 42.99], *SE* = 8.06, *z* = 5.07, *p* < 0.001). A smaller association was found for tabular representations (*OR* = 5.98, 95% CI [2.74, 13.05], *SE* = 2.38, *z* = 4.49, *p* < 0.001). The smallest, although still statistically significant association was observed for force diagram representations (*OR* = 2.84, 95% CI [1.57, 5.14], *SE* = 0.86, *z* = 3.46, *p* = 0.007).

Despite these associations, differences in success across target representation types persisted when intermediary representations were used. Problems requiring force diagram representations were associated with significantly lower success rates than those requiring table representations (*OR* = 0.15, 95% CI [0.07, 0.33], *SE* = 0.06, *z* = −4.60, *p* < 0.001), and mathematical representations (*OR* = 0.11, 95% CI [0.04, 0.32], *SE* = 0.06, *z* = −4.15, *p* < 0.001). No significant difference was observed between problems requiring tabular representations and mathematical representations.

### 3.2 Problem solving success across intermediary representation use and problem complexity types

To explore the interaction between intermediary representation use and problem complexity type, problem solving success was analyzed across problem complexity types and averaged across target representation types. Figure 2 presents success rates for each problem complexity type with and without intermediary representation use. When intermediary representations were not used, problems with force magnitude as the only unknown had significantly higher success rates than problems in which force magnitude and lever arm were unknown (*OR* = 47.57, 95% CI [26.53, 85.29], *SE* = 14.17, *z* = 12.97, *p* < 0.001), problems in which force magnitude and pivot were unknown (*OR* = 21.20, 95% CI [12.23, 36.75], *SE* = 5.95, *z* = 10.88, *p* < 0.001), and problems in which force magnitude and direction were unknown (*OR* = 13.40, 95% CI [7.79, 23.06], *SE* = 3.71, *z* = 9.39, *p* < 0.001). Success rates did not differ significantly between problems in which force magnitude and force direction were unknown and those in which force magnitude and pivot location were unknown (*OR* = 1.58, 95% CI [1.09, 2.29], *SE* = 0.30, *z* = 2.46, *p* = 0.211). However, both problem types had significantly higher success rates than problems in which force magnitude and lever arm length were unknown (*OR* = 3.55, 95% CI [2.36, 5.34], *SE* = 0.74, *z* = 6.11, *p* < 0.001; *OR* = 2.24, 95% CI [1.50, 3.35], *SE* = 0.46, *z* = 3.95, *p* = 0.002).

**Figure 2:**
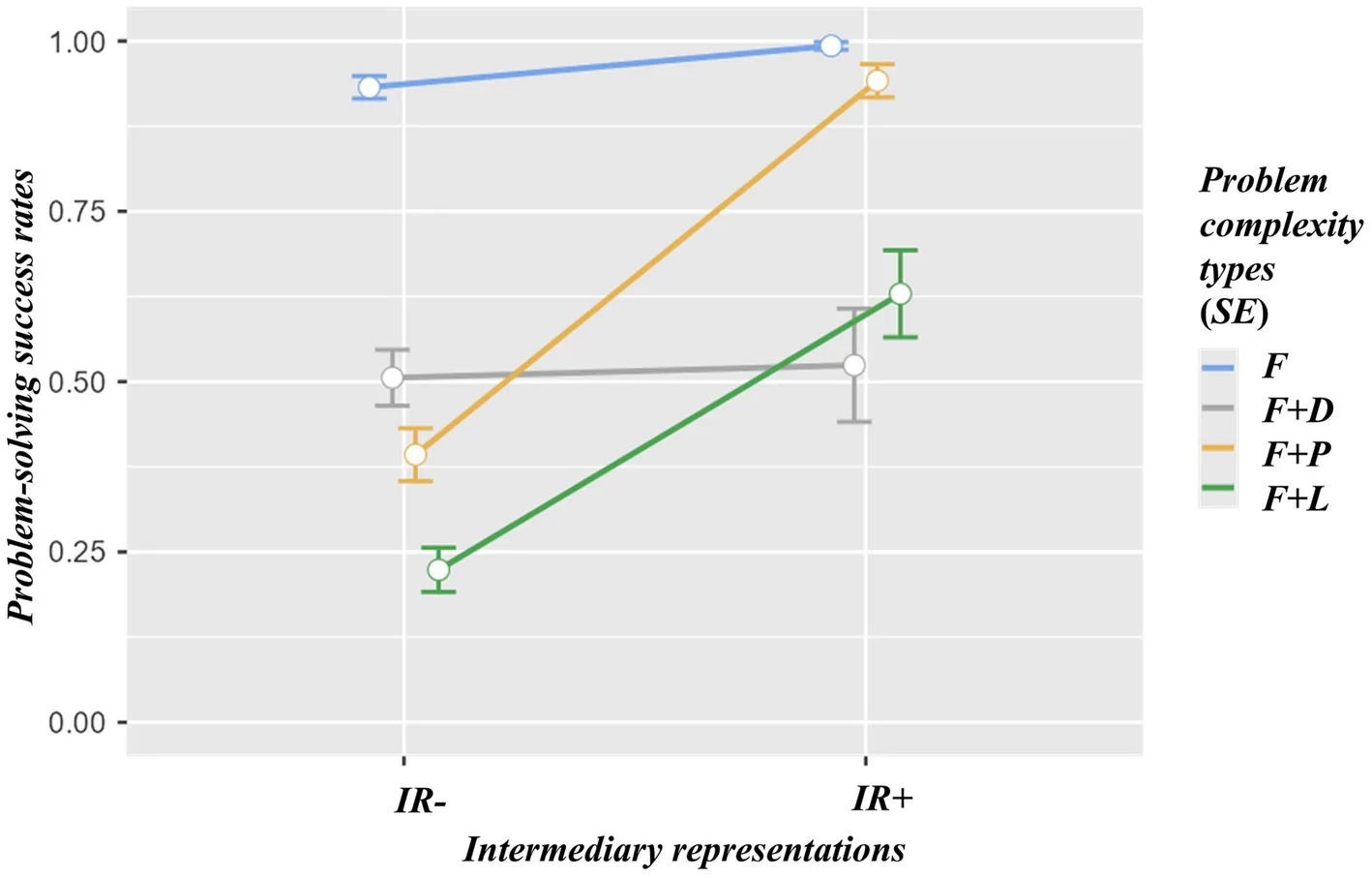
Problem-solving success rates across problem complexity types with and without intermediary representations. *F* = Force magnitude only; *F* + *P* = Force magnitude and pivot location; *F* + *D* = Force magnitude and force direction; *F* + *L* = Force magnitude and lever arm. IR− indicates no intermediary representation; IR+ indicates intermediary representation use. Error bars represent ± 1 standard error.

As shown in the upper panel of Table 3, the association between intermediary representation use and success varied across problem complexity types. Intermediary representation use was not significantly associated with success rates for problems with force magnitude as the only unknown (*OR* = 10.28, 95% CI [2.01, 52.48], *SE* = 8.55, *z* = 2.80, *p* = 0.094) or for problems in which both force magnitude and direction were unknown (*OR* = 1.08, 95% CI [0.54, 2.15], *SE* = 0.38, *z* = 0.21, *p* = 1.000). For problems in which force magnitude and lever arm were unknown, intermediary representation use was associated with significantly higher success rates (0.642 versus 0.261, *OR* = 5.88, 95% CI [3.23, 10.71], *SE* = 1.80, *z* = 5.79, *p* < 0.001). A significant association was also observed for problems in which force magnitude and pivot location were unknown, with success rates of 0.85 and 0.42, when intermediary representations were used and not used, respectively (*OR* = 24.96, 95% CI [10.14, 61.43], *SE* = 11.47, *z* = 7.00, *p* < 0.001).

When intermediary representations were used, problems with force magnitude as the only unknown continued to have the highest success rates. However, their success rates did not differ significantly from those of problems in which force magnitude and pivot location were unknown (*OR* = 8.74, 95% CI [1.59, 48.16], *SE* = 7.61, *z* = 2.49, *p* = 0.201). Success rates for both problem types were significantly higher than those for problems in which force magnitude and lever arm were unknown (*OR* = 83.19, 95% CI [16.01, 432.23], *SE* = 69.94, *z* = 5.26, *p* < 0.001 for force magnitude only; *OR* = 9.52, 95% CI [3.65, 24.85], *SE* = 4.66, *z* = 4.61, *p* < 0.001 for force magnitude and pivot location). Success rates for both problem types were also significantly higher than those for problems in which force magnitude and direction were unknown (*OR* = 128.04, 95% CI [24.22, 677.00], *SE* = 108.79, *z* = 5.71, *p* < 0.001 for force magnitude only; *OR* = 14.66, 95% CI [5.46, 39.38], *SE* = 7.39, *z* = 5.33, *p* < 0.001 for force magnitude and pivot location). No significant difference was observed between problems in which force magnitude and lever arm were unknown and those in which force magnitude and direction were (*OR* = 0.65, 95% CI [0.31, 1.38], *SE* = 0.25, *z* = 1.11, *p* = 0.956).

### 3.3 Problem solving success across target representation types and problem complexity types

Figure 3 illustrates success rates across target representation types within each problem complexity type. When only force magnitude was unknown, no significant differences were observed among force diagram, tabular, and mathematical representations (*OR* = 0.14, 95% CI [0.04, 0.49], *SE* = 0.09, *z* = −3.02, *p* = 0.101; *OR* = 0.54, 95% CI [0.20, 1.44], *SE* = 0.27, *z* = −1.22, *p* = 0.987; *OR* = 3.91, 95% CI [1.07, 14.25], *SE* = 2.58, *z* = 2.06, *p* = 0.651).

**Figure 3:**
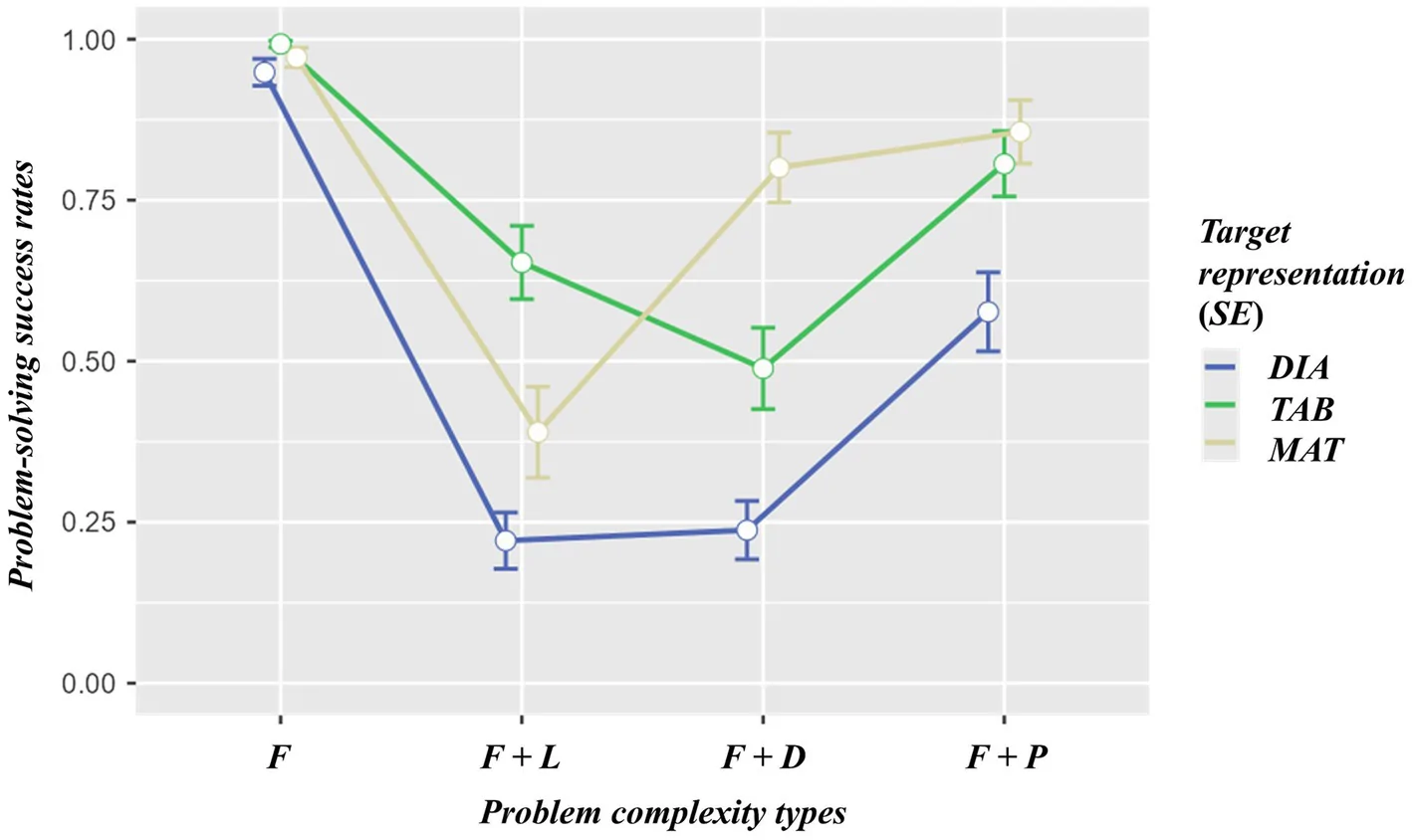
Problem-solving success rates by target representation and problem complexity types. TAB = tabular representation; MAT = mathematical representation; DIA = force diagram representation. Problem complexity types are coded as follows: *F* = force magnitude only; *F* + *P* = force magnitude and pivot location; *F* + *D* = force magnitude and force direction; *F* + *L* = force magnitude and lever arm. Error bars represent ±1 standard error.

However, for problems involving multiple unknowns, differences among target representation types were more pronounced. For problems in which force magnitude and pivot location were unknown, force diagram representations were associated with lower success rates than tabular representations, but this difference was not significant after Tukey adjustment (*OR* = 0.33, 95% CI [0.17, 0.63], *SE* = 0.11, *z* = −3.19, *p* = 0.064). However, force diagram representations were associated with significantly lower success rates than mathematical representations (*OR* = 0.23, 95% CI [0.11, 0.50], *SE* = 0.09, *z* = −3.59, *p* = 0.017), while no significant difference was observed between tabular and mathematical representations (*OR* = 0.70, 95% CI [0.31, 1.58], *SE* = 0.29, *z* = −0.87, *p* = 0.999). For problems in which force magnitude and lever arm were unknown, force diagram representations were associated with significantly lower success rates than tabular representations (*OR* = 0.15, 95% CI [0.08, 0.29], *SE* = 0.05, *z* = −5.80, *p* < 0.001). Mathematical representations fell between the two, with no significant differences relative to either tabular or force diagram representations (*OR* = 0.45, 95% CI [0.22, 0.90], *SE* = 0.16, *z* = −2.25, *p* = 0.510; *OR* = 2.95, 95% CI [1.46, 5.97], *SE* = 1.06, *z* = 3.02, *p* = 0.104). For problems in which force magnitude and direction were unknown, force diagram representations were associated with significantly lower success rates than tabular representations (*OR* = 0.33, 95% CI [0.18, 0.60], *SE* = 0.10, *z* = −3.58, *p* = 0.018) and mathematical representations (*OR* = 0.08, 95% CI [0.04, 0.17], *SE* = 0.03, *z* = −6.73, *p* < 0.001). Tabular representations were associated with significantly lower success rates than mathematical representations (*OR* = 0.24, 95% CI [0.12, 0.50], *SE* = 0.09, *z* = −3.82, *p* = 0.007).

### 3.4 Within and between student sensitivity analysis

A sensitivity analysis examined whether the association between additional representation use and problem solving success was observable within the same students across items. The model converged without warnings and showed no evidence of overdispersion, with a dispersion ratio of 0.779. The participant random intercept variance was 1.058, corresponding to an ICC of 0.243, and a likelihood ratio test supported retaining the participant random intercept, *χ*^2^(1) = 94.38, *p* < 0.001.

The within student component was significantly associated with problem solving success, *β* = 1.32, *SE* = 0.19, *OR* = 3.74, 95% CI [2.56, 5.46], *p* < 0.001. After controlling for item identity and students’ general propensity to use additional representations, students had higher odds of success on items for which an additional representation was observed than on items for which one was not observed. The between student component was also significant, *β* = 2.230, *SE* = 0.512, *OR* = 9.30, 95% CI [3.41, 25.38], *p* < 0.001. Item identity was also significant, *χ*^2^(11) = 262.59, *p* < 0.001, indicating substantial performance variation among the administered items.

An algebraically equivalent reparameterization showed that the difference between the within student and between student coefficients was not statistically significant, $\beta_{\text{differnece}}$ = 0.91, *SE* = 0.54, *OR* = 2.49, 95% CI [0.87, 7.12], *p* = 0.088. Thus, there was no statistically reliable evidence that the magnitudes of the within student and between student associations differed.

## 4 Discussion

### 4.1 Intermediary representations and problem solving

The results showed a robust association between intermediary representations use and problem solving success in lever balance tasks. Across problem types and target representation types, responses accompanied by intermediary representations had substantially higher success rates than responses without them. However, because students generated these representations spontaneously rather than being assigned to use them, the present design does not establish that intermediary representation construction caused higher success rates.

From a disciplinary perspective, representations are not interchangeable formats but tools that selectively foreground particular aspects of physical meaning while backgrounding others. As articulated by Fredlund et al. (2014), different representations possess different disciplinary affordances. Some representations are more suitable for expressing quantitative relations, whereas others better express spatial or structural relations. In the present study, the source representations provided access to certain aspects of the lever system, yet did not always make all physically relevant relations equally salient. One possible interpretation is that students who constructed intermediary representations were able to make insufficiently salient relations more explicit. For example, constructing a schematic diagram, annotating distances, or writing an intermediate equation may make explicit the relational structure required for equilibrium reasoning. The observed association may therefore reflect more effective access to disciplinary meaning through representational reconfiguration, although this process was not directly measured.

The findings also resonate with research on representational translation. Adu-Gyamfi et al. (2012) identified three major sources of difficulty in representational work: misinterpretation of the source representation, failure to preserve relational structure during translation, and procedural or implementation errors in constructing the target representation. Accordingly, one possible explanation for the present pattern is that externalizing relational structure before target construction reduced opportunities for structural distortion or helped organize quantities before symbolic manipulation.

Another possible interpretation of the observed association draws on metacognitive and generative learning accounts. By externalizing intermediate relations, students may create opportunities to inspect the coherence of their reasoning, compare emerging representations with physical constraints, and regulate subsequent problem solving processes (Kohl and Finkelstein, 2006; Renkl et al., 2013; Álvarez et al., 2020; Sijmkens et al., 2023). From a generative learning perspective, actively constructing an intermediary representation may prompt learners to select relevant information, organize relational elements, and integrate them into a coherent external representation (Fiorella and Mayer, 2016; Fiorella and Zhang, 2018; van Meter and Garner, 2005; Wu and Rau, 2019). Such construction may also create opportunities for self-explanation by requiring learners to externalize relations that might otherwise remain implicit (Berthold et al., 2009; Chi et al., 1994). In this sense, intermediary representations function not only as structural bridges between source and target formats, but also as generative cognitive tools that support self-explanation and conceptual checking. Although such processes do not guarantee conceptual revision, they may reduce the persistence of unexamined structural and procedural errors during subsequent symbolic manipulation (Adu-Gyamfi et al., 2012). However, metacognitive monitoring and generative processing were not directly assessed in the present study. These accounts should therefore be treated as theoretically plausible explanations rather than mechanisms demonstrated by the data.

The higher success rates associated with intermediary representation use also resemble representational practices documented in research on expert and novice physics problem solving. Expert problem solvers often construct intermediate qualitative or schematic representations before a formal mathematical solution, whereas novices are more likely to rely directly on familiar surface forms or equations (Kohl and Finkelstein, 2006, 2008). Students who construct intermediary representations may therefore use strategies similar to those documented among more expert solvers. However, because the present coding captured only the occurrence of an intermediary representation and not its quality, timing, or strategic intent, intermediary representation use should not by itself be interpreted as evidence of emerging expertise or representational sophistication.

### 4.2 Conditional associations across target representation and problem complexity

Beyond the overall association between intermediary representation use and problem solving success, the strength of this association varied across target representation types and problem complexity types. These interactions indicate conditional patterns, although their interpretation remains tentative.

With respect to target representation type, the largest performance difference associated with intermediary representation use was observed when the target format was mathematical, followed by tabular and force diagram target representations. At the same time, force diagram targets were associated with lower overall success rates across complexity types. One possible interpretation draws on the representational chain model (Johri et al., 2013), which conceptualizes problem solving as movement along a continuum between experiential or spatially grounded representations and abstract symbolic forms. Transformations along this continuum require preserving relational structure while changing representational form. In the present context, mathematical targets occupy a more abstract position along this continuum. Translating a spatial lever configuration into a symbolic equilibrium equation requires perceptual information to be reorganized into algebraic relations. This transformation demands structural compression and abstraction. Under such conditions, constructing an intermediary representation, particularly a diagrammatic or structured relational form, may serve as a stabilizing intermediate link in the representational chain. Rather than translating directly from the source situation to a symbolic equation, students can first externalize spatial relations, identify lever arms, and organize force directions before formalization. This may reduce the amount of representational restructuring required in a single step. This interpretation may help explain why the largest difference associated with intermediary representation use was observed when the target was mathematical. By contrast, when the target representation was itself a diagram, an additional representation may provide less structural reorganization beyond what the task already requires. Because the sequence of representational transformations was not directly observed, this account remains a theoretical interpretation.

Related evidence comes from Chen et al. (2017), who showed that externally provided diagrams do not always improve performance and may reduce learners’ tendency to construct their own representations. When a diagram is already given or required as the endpoint, learners may engage less in generative structural organization. In contrast, when the final goal is symbolic or tabular, diagram construction may function as a productive intermediary step that supports relational encoding prior to abstraction. More broadly, research on learner generated drawing indicates that representation construction does not uniformly improve learning. Its contribution depends on factors such as the comparison activity, the guidance provided, the learner’s prior knowledge, and the correspondence between the drawing activity and the cognitive demands of the task (Fiorella and Zhang, 2018; van Meter and Garner, 2005; Wu and Rau, 2019). In the present study, one possible interpretation is that mathematical and tabular targets created a greater need to reorganize spatial information into abstract or structured relations, allowing an intermediary diagram or schematic representation to serve as an additional organizational step. When the required target was already diagrammatic, the function of an intermediary representation may have overlapped more closely with that of the target representation, resulting in a smaller incremental association with problem solving success.

The interaction between intermediary representation use and target representation should be interpreted as variation in the observed association across target specific task conditions. Problem solving success was evaluated using criteria appropriate to each required response product. Mathematical responses, tabular responses, and force diagram responses therefore differed not only in representational form but also in the components that students were required to produce and the criteria used to determine successful completion. Consequently, the differences observed across these conditions cannot be attributed solely to representation format. They may also reflect differences in response requirements, production demands, and scoring criteria.

The association between intermediary representation use and problem solving success also varied across problem complexity types. The largest differences were observed for problems requiring coordination of force magnitude with pivot location or lever arm length, whereas smaller or nonsignificant differences occurred for problems involving force magnitude only or force magnitude and direction. This was a category specific pattern across the administered problems, not evidence of a general nonlinear relationship in which intermediary representations are most beneficial at a moderate level of complexity. The expert ratings provided only partial support for distinctions among the three multiple unknown types. Moreover, intermediary representation use was observational, and each target representation by complexity cell contained one item. The interaction pattern should therefore be interpreted tentatively.

This interaction pattern may be interpreted through the distinction between content related and processing related demands articulated by Lee and Heyworth (2000). Problems involving multiple unknowns required students to coordinate multiple interacting quantities, integrate geometric or relational constraints, and infer intermediate quantities prior to applying the equilibrium condition. Under such circumstances, intermediary representations may have helped externalize partial relations and supported more sequential coordination of information. This possibility is consistent with the composition effect literature, which shows that performance declines when multiple relations must be processed concurrently rather than sequentially (Heffernan and Koedinger, 1997), and with evidence showing that simultaneous integration imposes greater demands than stepwise resolution (Ibrahim et al., 2017). However, working memory demands and the temporal sequence of students’ reasoning were not directly measured.

However, the pattern also suggests boundary conditions. For problems involving force magnitude only, intermediary representation use showed little additional association with success. When only force magnitude was unknown, the relevant force directions and lever arms were already available in the source representation, and the equilibrium relation could be applied directly. One possible explanation is the redundancy effect in cognitive load theory, according to which additional information may provide little benefit when it duplicates information that is already explicit or readily accessible (Chandler and Sweller, 1991; Sweller, 1988). It is also consistent with the DeFT framework, which proposes that multiple representations are beneficial when they serve identifiable functions, such as complementing information, constraining interpretation, or supporting construction (Ainsworth, 2006). Because cognitive load was not measured, the redundancy interpretation remains tentative.

A different possible boundary condition concerns problems involving both force magnitude and force direction. These problems required students to infer the direction of the unknown force, identify the corresponding line of action, determine the relevant lever arm, and then apply the equilibrium condition. From a cognitive load perspective, these mutually dependent elements may create high element interactivity (Sweller, 1988; Sweller et al., 1998; van Merriënboer and Sweller, 2005). Constructing and coordinating an additional representation also requires attentional and working memory resources (Lee et al., 2009). Thus, one possible explanation is that the additional construction demand partly offset the structural support provided by the intermediary representation. Research on multiple representations similarly indicates that learners must select, organize, and map information across forms and that poorly coordinated representations can interfere with coherent mental model construction (Ainsworth, 2006; Schnotz and Bannert, 2003; Seufert, 2003). This pattern is consistent with possible representational overload, but cognitive load and coordination processes were not directly assessed.

Taken together, the category specific differences are consistent with the possibility that the association between intermediary representation use and success varies according to the relational and processing demands of a problem. This explanation remains tentative and requires direct testing through experimental manipulation, replicated items, and measures of cognitive load, representational sequencing, and strategy use.

### 4.3 Implications

The present findings carry several implications for physics instruction in problem solving contexts. First, teachers may consider encouraging students to construct intermediary representations when tasks require substantial relational reorganization, particularly when the required target is mathematical or tabular. In such contexts, diagrams generated by students may serve as transitional tools for organizing spatial relations before formalization.

Second, the observed pattern also suggests that representational prompts may need to be adapted to problem complexity rather than imposed uniformly. For simple problems, an additional representational step may provide little added value. For highly demanding problems, students may require guidance in identifying force directions or lever arms before independently generating a useful intermediary representation. Representational prompting may therefore be most promising for problems that require meaningful but manageable relational coordination, such as pivot identification or lever arm inference. This instructional proposal should be tested directly in future intervention research.

Third, instruction may also focus on helping students decide why and when to construct an intermediary representation rather than treating representation production as a fixed procedural requirement. Existing research suggests that drawing prompts, representational guidance, and self-explanation support can facilitate productive generative activity (Ainsworth et al., 2011; Berthold et al., 2009; Fiorella and Zhang, 2018). Nevertheless, because the present study recorded the occurrence of intermediary representations rather than their quality or associated cognitive processes, these instructional implications remain provisional.

### 4.4 Limitations

Several limitations should be considered when interpreting the present findings. First, the generalizability of the findings is limited by both the participant sample and the content domain. Participants were 130 ninth grade students from three classes taught by one teacher at one school in one city. Because test submission was voluntary and anonymous, respondents could not be compared with nonrespondents at the individual level, and possible selection differences cannot be ruled out. In addition, the study examined only lever balance problems, and the observed patterns may not extend to other physics domains with different representational conventions and problem solving demands. Future studies should replicate these findings across more diverse student populations and content domains.

Second, each target representation by problem complexity cell was represented by only one item, making item specific features inseparable from the intended conditions. Models containing participant and item random intercepts simultaneously did not converge. When estimated separately, participant level variability was substantially greater than item level variability, supporting the retention of the participant random intercept in the final model. Because item variability was not modelled, the findings should be interpreted as patterns among the administered items rather than as effects that necessarily generalize to the broader population of lever balance problems. Future studies should include multiple items within each condition cell to estimate participant and item variability simultaneously.

Third, the study focused exclusively on students’ self-constructed intermediary representations. However, prior knowledge was not experimentally controlled. From a generative learning perspective, the relationship between constructing intermediary representations and problem solving success may vary according to learners’ existing knowledge (Fiorella and Mayer, 2016). Students with higher prior knowledge may be better able to generate structurally appropriate intermediary representations, whereas students with lower prior knowledge may require more guided support. Future research should therefore investigate how prior knowledge relates to students’ likelihood of constructing intermediary representations, the quality of those representations, and their association with problem solving success.

Fourth, the operational indicator of intermediary representation use was target dependent and encompassed heterogeneous representational forms. A particular representational form could qualify as additional in one target condition while constituting the required response in another. Consequently, the prevalence and composition of the coded variable differed across target conditions. Moreover, the coding captured the observable occurrence of an additional non target representation rather than its quality, sophistication, accuracy, completeness, temporal position, or cognitive function. Partial or incorrect additional representations were coded as present, and some occurred when the required target response was incomplete, incorrect, or absent. The present data therefore do not establish that an additional representation preceded, mediated, or facilitated target production, nor do they distinguish productive representations from superficial or potentially misleading ones. Future studies should combine written responses with fine grained qualitative or polytomous coding of representational accuracy, coherence, completeness, and function, as well as process measures such as think aloud protocols, digital trace data, or sequential analyses.

Fifth, intermediary representation use was observed rather than experimentally assigned. Students who constructed intermediary representations may have differed from other students in prior knowledge, representational competence, motivation, or strategy selection, any of which could also contribute to problem solving success. Moreover, metacognitive monitoring, self-explanation, generative processing, and cognitive load were not directly assessed. Accordingly, these constructs should be treated as possible explanatory mechanisms rather than mediators established by the present data. Future studies should experimentally manipulate representational prompts, measure relevant student characteristics, and directly assess the proposed cognitive processes to evaluate their respective contributions to problem solving performance.

Finally, the criteria for problem solving success were aligned with the designated target representation. Mathematical, tabular, and force diagram responses consequently differed in both representational form and the specific components required for successful completion. Differences among target conditions, including the interaction between intermediary representation use and target representation, should therefore be interpreted as differences in target specific task performance rather than as pure effects of representation format. Future studies could use parallel response requirements and common scoring dimensions across target conditions to distinguish the contribution of representational format from other task requirements and scoring criteria.

## 5 Conclusion

This study examined the relationship between intermediary representation use and success in lever balance problem solving and investigated how this relationship varied across target representation types and problem complexity types. Intermediary representation use was associated with higher overall problem solving success. The association was strongest for mathematical or tabular target representation and more limited for diagrammatic target representations. Its magnitude also varied across problem complexity types. Larger differences were observed for problems requiring coordination of force magnitude with pivot location or lever arm length, whereas smaller or nonsignificant differences occurred for problems involving force magnitude only or force magnitude and direction. Because intermediary representation use was observed rather than experimentally assigned, these findings do not demonstrate that constructing an intermediary representation caused higher success. The patterns are consistent with the possibility that representational demands and problem complexity are associated with the strength of the observed relationship.

## Data Availability

The raw data supporting the conclusions of this article will be made available by the authors, without undue reservation.
