# Supplementary material for: The role of intermediary representations in solving lever balance problems with different target representations and problem complexities
Source: Front Psychol. 2026 Sep 17;17:1815951. doi: 10.3389/fpsyg.2026.1815951 (PMC13627041; doi:10.3389/fpsyg.2026.1815951)
Supplement: Supplementary file 1 [file Data_Sheet_1.pdf]

Supplementary Figure S1. Examples illustrating the independent coding of problem-solving success and intermediary representation use.

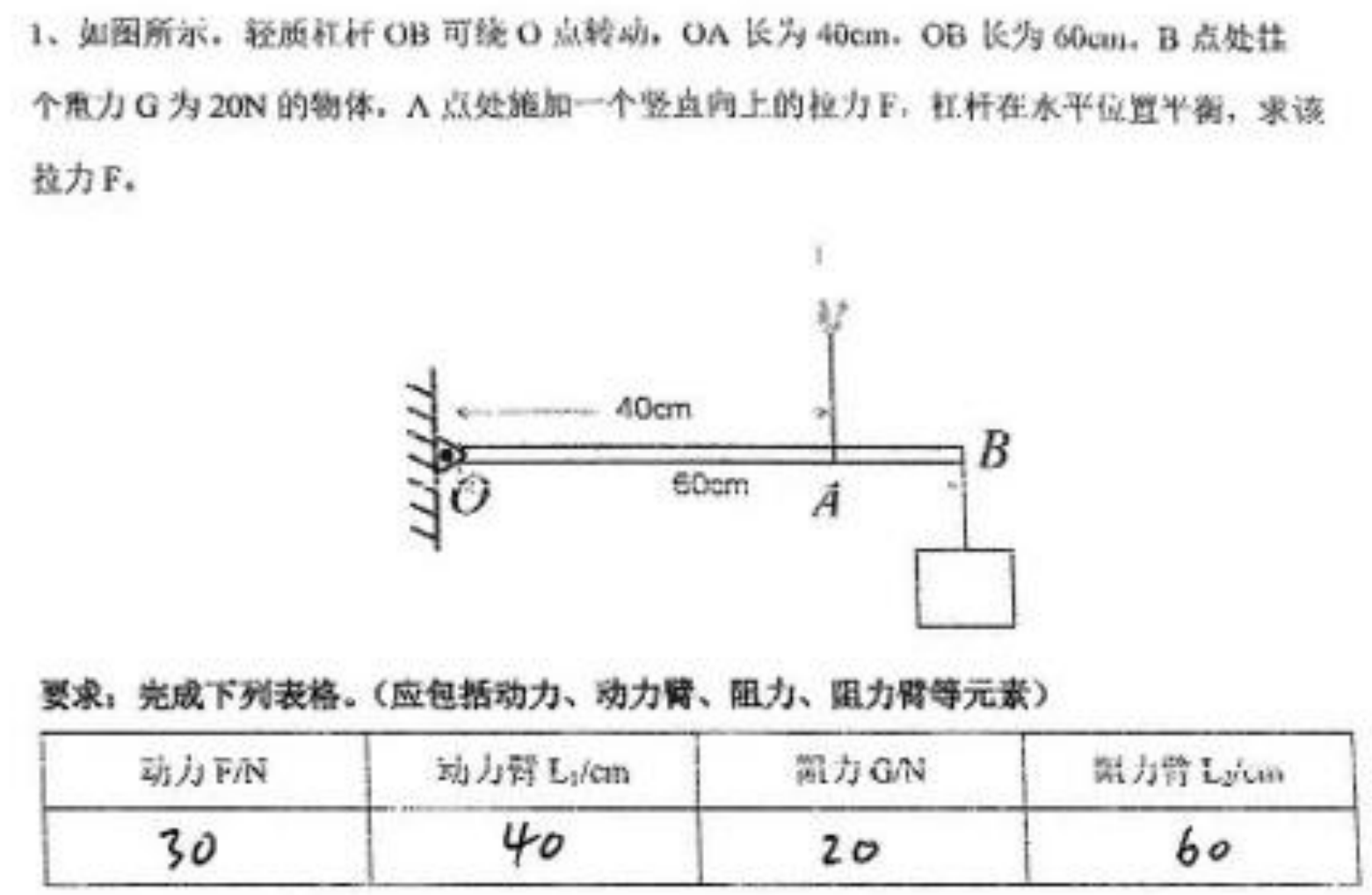

1、如图所示，轻质杠杆 OB 可绕 O 点转动，OA 长为 40cm，OB 长为 60cm，B 点处挂个重力 G 为 20N 的物体，A 点处施加一个竖直向上的拉力 F，杠杆在水平位置平衡，求该拉力 F。

要求：完成下列表格。（应包括动力、动力臂、阻力、阻力臂等元素）

动力 F/N	动力臂 L_1/cm	阻力 G/N	阻力臂 L_2/cm
30	40	20	60

(A) Successful response without intermediary representation use (PS = 1, IR = 0). The designated target was tabular. The student correctly entered F = 30 N, L1 = 40 cm, G = 20 N, and L2 = 60 cm without constructing an additional representation.

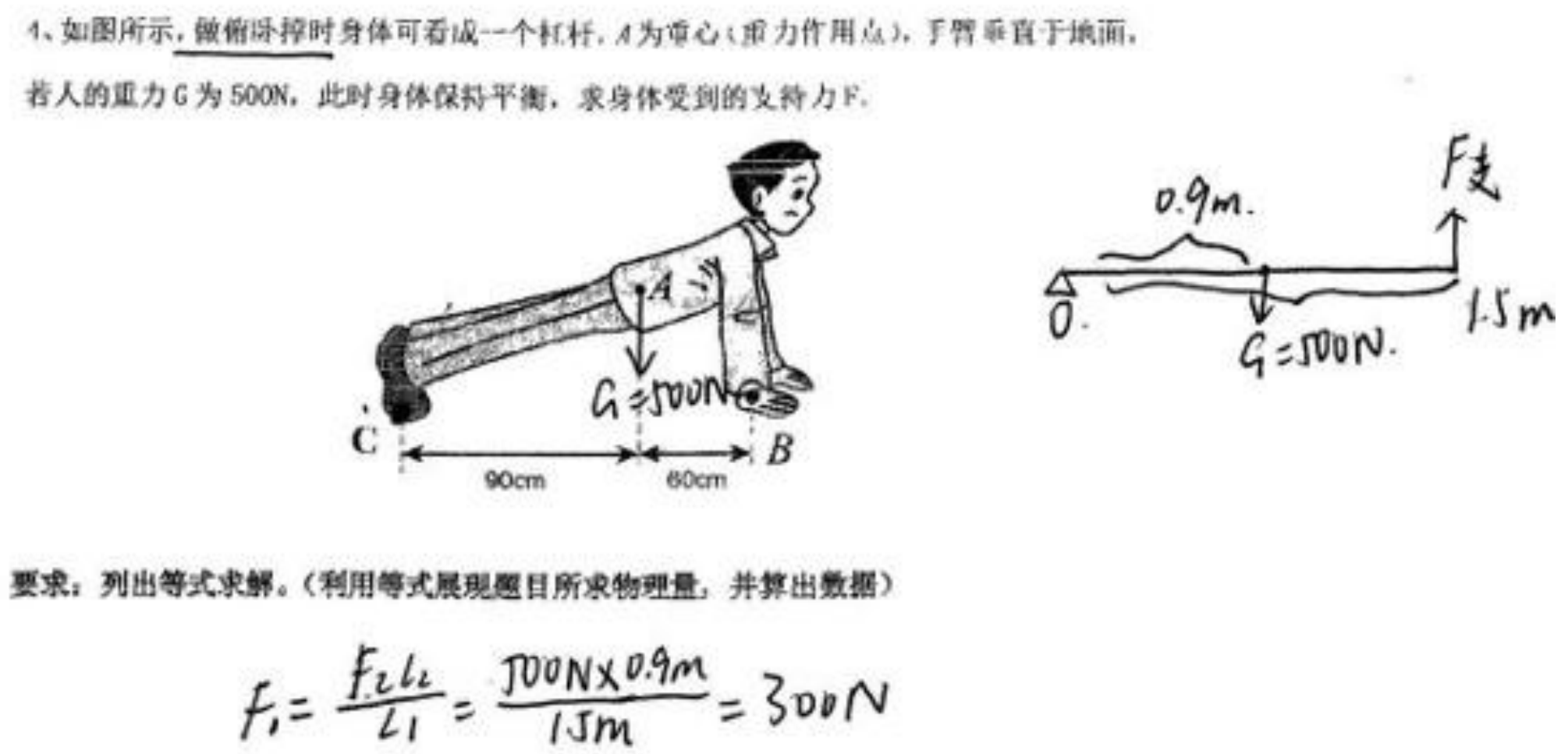

4、如图所示，做俯卧撑时身体可看成一个杠杆，A 为重心（重力作用点），手臂垂直于地面。若人的重力 G 为 500N，此时身体保持平衡，求身体受到的支持力 F。

要求：列出等式求解。（利用等式展现题目所求物理量，并算出数据）

$$F_1 = \frac{F_2 L_2}{L_1} = \frac{500N \times 0.9m}{1.5m} = 300N$$

(B) Successful response with intermediary representation use (PS = 1, IR = 1). The designated target was mathematical. Before completing the equation, the student constructed an additional lever diagram showing the pivot, forces, and moment arms and then obtained the correct result, F = 300 N.

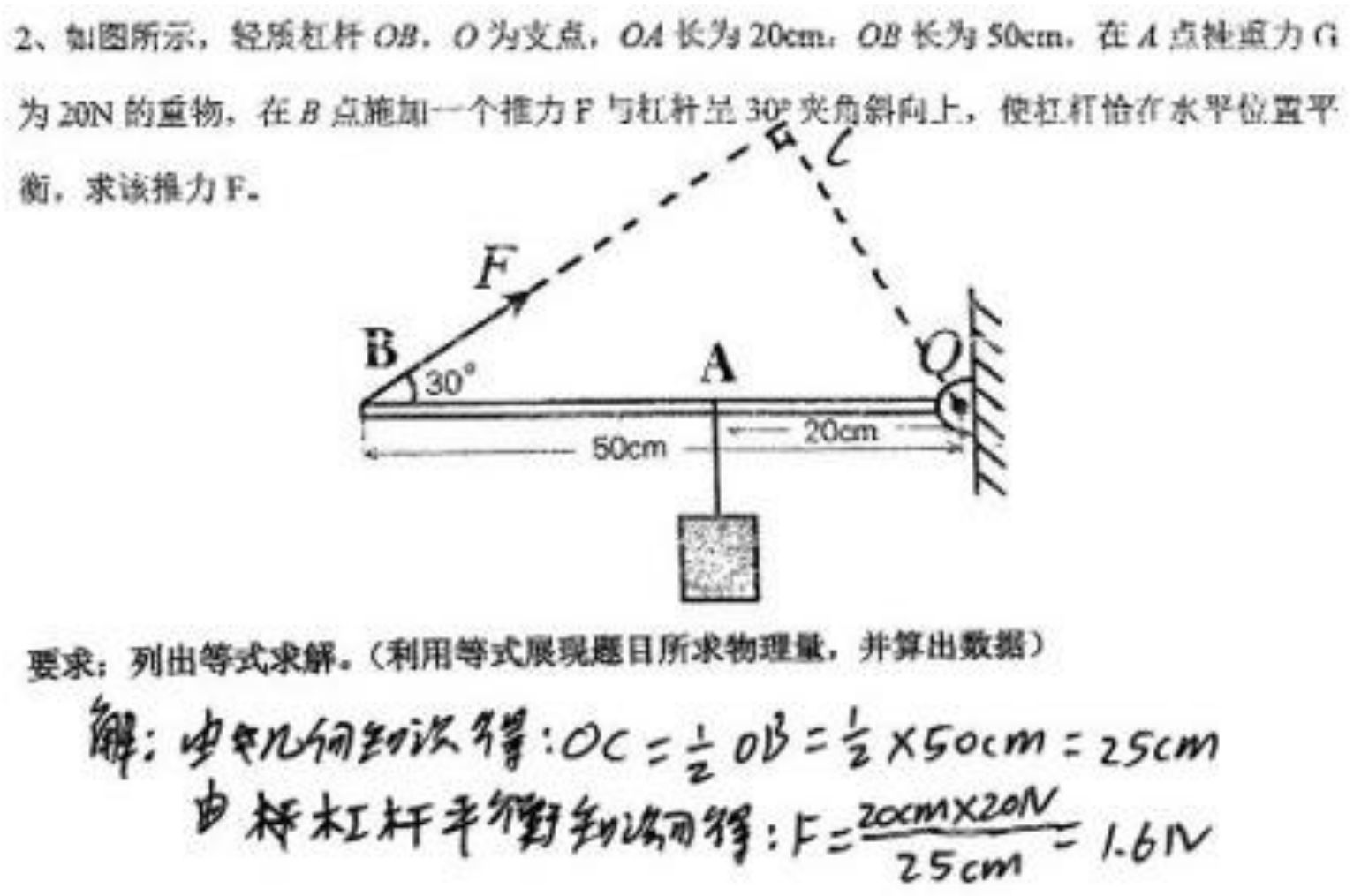
2、如图所示，轻质杠杆 OB，O 为支点，OA 长为 20cm，OB 长为 50cm，在 A 点挂重力 G 为 20N 的重物，在 B 点施加一个推力 F 与杠杆呈 30° 夹角斜向上，使杠杆恰在水平位置平衡，求该推力 F。

要求：列出等式求解。（利用等式展现题目所求物理量，并算出数据）

解：由几何知识得：$OC = \frac{1}{2} OB = \frac{1}{2} \times 50cm = 25cm$

由杠杆平衡知识可得：$F = \frac{20cm \times 20N}{25cm} = 1.6N$

(C) Unsuccessful response with intermediary representation use (PS = 0, IR = 1). The designated target was mathematical. The student added an auxiliary geometric construction comprising point C, perpendicular line OC, and a right-angle marker to identify the moment arm. This construction was coded as intermediary representation use, but the response was unsuccessful because 400/25 was calculated as 1.6 N rather than 16 N.

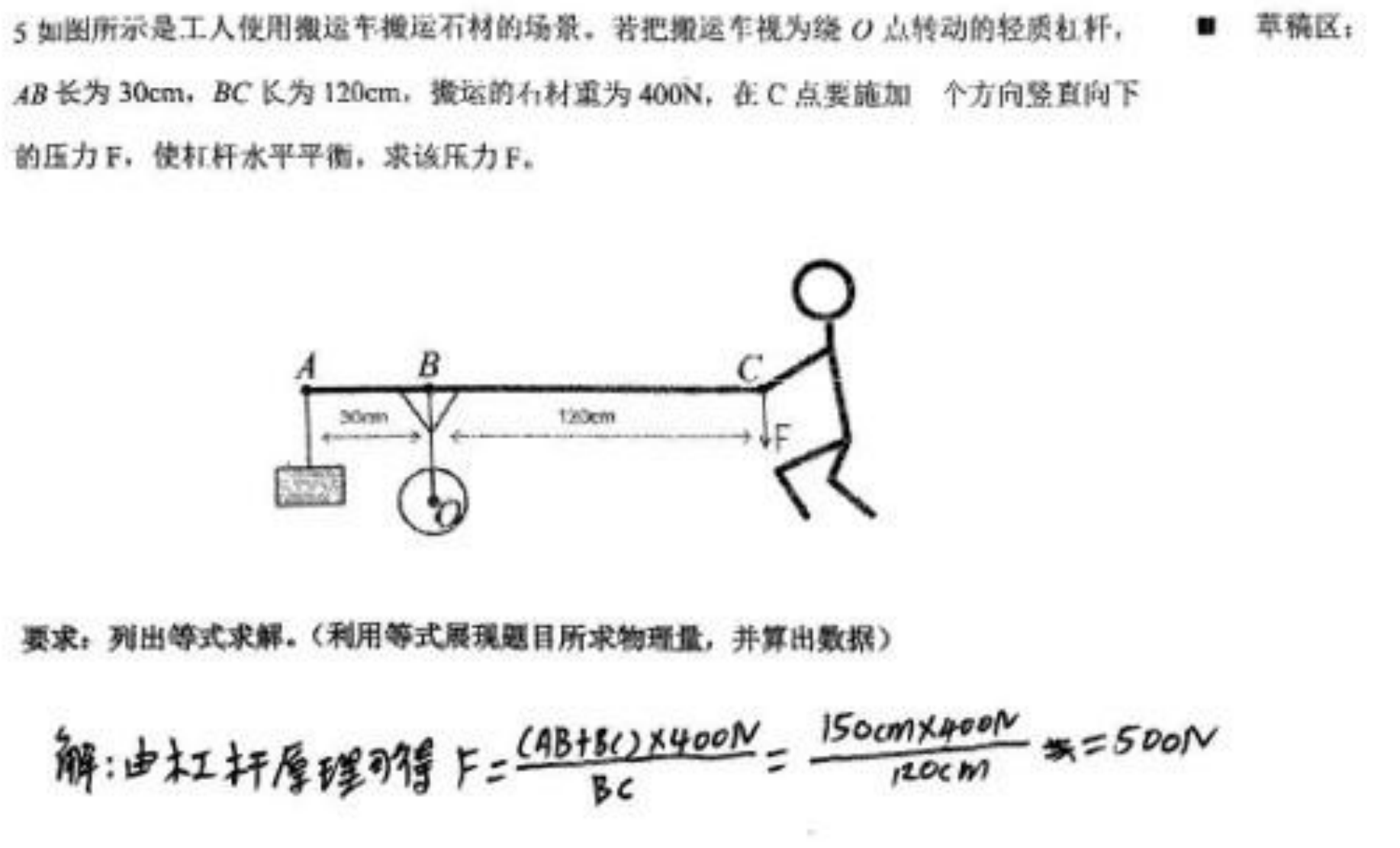
5 如图所示是工人使用搬运车搬运石材的场景。若把搬运车视为绕 O 点转动的轻质杠杆，AB 长为 30cm，BC 长为 120cm，搬运的石材重为 400N，在 C 点要施加 个方向竖直向下的压力 F，使杠杆水平平衡，求该压力 F。

■ 草稿区：

要求：列出等式求解。（利用等式展现题目所求物理量，并算出数据）

解：由杠杆原理可得 $F = \frac{(AB+BC) \times 400N}{BC} = \frac{150cm \times 400N}{120cm} = 500N$

(D) Unsuccessful response without intermediary representation use (Question 5; PS = 0, IR = 0). The designated target was mathematical, and the student used only the required equation format. The response was unsuccessful because point B should be treated as the pivot and AB = 30 cm as the load arm, giving F = 100 N rather than 500 N.

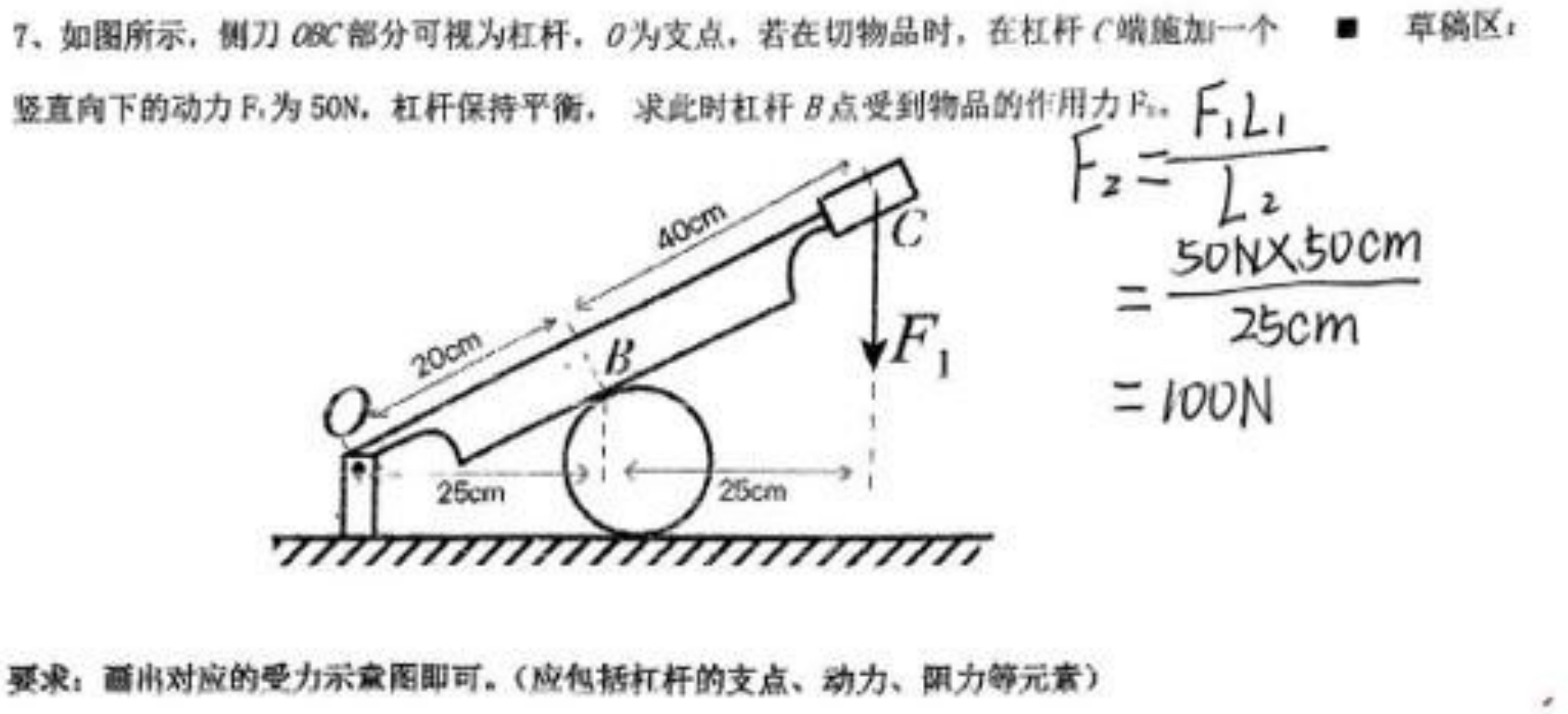

(E) Unsuccessful response with an incorrect intermediary representation (PS = 0, IR = 1). The designated target was a force diagram. The student omitted the required diagram and instead constructed an additional mathematical representation. The equation used 25 cm rather than 20 cm as the resistance arm, yielding 100 N rather than 125 N. The representation was nevertheless coded as present because occurrence was coded independently of accuracy.

Note. PS denotes problem-solving success, and IR denotes intermediary representation use. The variables were coded independently at the item level. PS was coded 1 when the response accurately completed the designated target representation and correctly expressed the relevant physical relationships, including the final force magnitude when required; otherwise, PS was coded 0. IR was coded 1 when the student constructed an optional, task-relevant external representation beyond the designated target, such as an additional diagram, table, equation, auxiliary geometric construction, or meaningful annotation to the supplied image that identified or reorganized forces, directions, pivot locations, lines of action, moment arms, or other relations. A representation required by the target was not counted as intermediary use. Thus, an equation written for a mathematical target was part of the target response, whereas an equation could be coded as intermediary use for a tabular or diagrammatic target. Simple copying, circling, underlining, isolated numerical work, illegible or unrelated marks, and clearly abandoned or crossed-out work were not counted. Partial or incorrect representations were coded as present when their task-relevant representational intent was identifiable; their quality did not determine the binary IR code.
