# Supplementary material for: The role of intermediary representations in solving lever balance problems with different target representations and problem complexities
Source: Front Psychol. 2026 Sep 17;17:1815951. doi: 10.3389/fpsyg.2026.1815951 (PMC13627041; doi:10.3389/fpsyg.2026.1815951)
Supplement: Supplementary file 2 [file Table_1.PDF]

Supplementary Table S1. Distribution of coded additional non-target representations across target representation conditions

Target representation	Additional non-target representations use n (%)		
	Force diagram	Mathematical	Tabular
Force diagram	Not applicable	168 (32.3%)	0
Mathematical	68 (13.1%)	Not applicable	0
Tabular	32 (6.2%)	94 (18.1%)	Not applicable
Total	100 (6.4%)	262 (16.8%)	0

Note. Percentages for individual target representation conditions were calculated using the 520 responses in each condition. Percentages in the total row were calculated using all 1,560 responses. "Not applicable" indicates that the representation was required as the designated target and therefore was not eligible to be coded as an additional non-target representation. A force diagrammatic representation comprised student-generated force or lever representations, including a separately drawn force diagram, an auxiliary geometric construction used to determine a force relation or lever arm, or task-relevant force and distance annotations added directly to the supplied schematic. The supplied schematic itself was not coded as an additional representation. As illustrated in Figure S1(C), student-generated force information added to the supplied schematic could be coded as an additional force diagrammatic representation when the required target was mathematical or tabular. A mathematical representation comprised student-generated equations or symbolic calculations when mathematics was not the required target. No additional tabular representations were observed. Because the written responses did not establish the order in which representations were produced, these categories indicate the observable presence of additional non-target representations rather than demonstrating that they temporally mediated the production of the required target representation.
