# Supplementary material for: The role of intermediary representations in solving lever balance problems with different target representations and problem complexities
Source: Front Psychol. 2026 Sep 17;17:1815951. doi: 10.3389/fpsyg.2026.1815951 (PMC13627041; doi:10.3389/fpsyg.2026.1815951)
Supplement: Supplementary file 3 [file Table_2.PDF]

Supplementary Table S2. Problem solving success and additional representation use across the 12 items

Item	Problem complexity	Target representation	IR absent		IR present	
			n	Successful, *n* (%)	*n*	Successful, *n* (%)
1	Force magnitude only	Force diagram	78	64 (82.1%)	52	52 (100.0%)
2	Force magnitude only	Tabular	96	92 (95.8%)	34	34 (100.0%)
3	Force magnitude only	Mathematical	116	98 (84.5%)	14	12 (85.7%)
4	Force magnitude and lever arm length	Force diagram	90	18 (20.0%)	40	14 (35.0%)
5	Force magnitude and lever arm length	Tabular	90	40 (44.4%)	40	32 (80.0%)
6	Force magnitude and lever arm length	Mathematical	104	16 (15.4%)	26	22 (84.6%)
7	Force magnitude and direction	Force diagram	92	30 (32.6%)	38	10 (26.3%)
8	Force magnitude and direction	Tabular	106	52 (49.1%)	24	12 (50.0%)
9	Force magnitude and direction	Mathematical	116	78 (67.2%)	14	12 (85.7%)
10	Force magnitude and pivot location	Force diagram	92	32 (34.8%)	38	28 (73.7%)
11	Force magnitude and pivot location	Tabular	102	48 (47.1%)	28	26 (92.9%)
12	Force magnitude and pivot location	Mathematical	116	50 (43.1%)	14	14 (100.0%)
	**Total**		**1,198**	**618 (51.6%)**	**362**	**268 (74.0%)**

Note. IR absent indicates that no additional non-target representation was observed, whereas IR present indicates that at least one additional non-target representation was observed. Each item was completed by 130 students.
